# Epidermal Growth Factor in the CNS: A Beguiling Journey from Integrated Cell Biology to Multiple Sclerosis. An Extensive Translational Overview

**DOI:** 10.1007/s10571-020-00989-x

**Published:** 2020-11-05

**Authors:** Giuseppe Scalabrino

**Affiliations:** Department of Biomedical Sciences, University of Milan, Via Mangiagalli 31, 20133 Milan, Italy

**Keywords:** Epidermal growth factor, Experimental CNS demyelinating diseases, Multiple sclerosis, Myelin, Neural stem cells, Oligodendrocytes

## Abstract

This article reviews the wealth of papers dealing with the different effects of epidermal growth factor (EGF) on oligodendrocytes, astrocytes, neurons, and neural stem cells (NSCs). EGF induces the in vitro and in vivo proliferation of NSCs, their migration, and their differentiation towards the neuroglial cell line. It interacts with extracellular matrix components. NSCs are distributed in different CNS areas, serve as a reservoir of multipotent cells, and may be increased during CNS demyelinating diseases. EGF has pleiotropic differentiative and proliferative effects on the main CNS cell types, particularly oligodendrocytes and their precursors, and astrocytes. EGF mediates the in vivo myelinotrophic effect of cobalamin on the CNS, and modulates the synthesis and levels of CNS normal prions (PrP^C^s), both of which are indispensable for myelinogenesis and myelin maintenance. EGF levels are significantly lower in the cerebrospinal fluid and spinal cord of patients with multiple sclerosis (MS), which probably explains remyelination failure, also because of the EGF marginal role in immunology. When repeatedly administered, EGF protects mouse spinal cord from demyelination in various experimental models of autoimmune encephalomyelitis. It would be worth further investigating the role of EGF in the pathogenesis of MS because of its multifarious effects.

## Introduction and Historical Benchmarks

Epidermal growth factor (EGF) was first isolated from mouse submaxillary glands (Cohen 1962). In relation to the central nervous system (CNS), the first demonstration of the EGF presence in human cerebrospinal fluid (CSF) was in 1982 (Hirata et al. 1982), the first evidence of EGF cross˗reacting material in rat brain was provided in 1984 (Fallon et al. 1984), and the presence of EGF mRNA in the mouse brainstem and striatum was shown in 1992 (Lazar and Blum 1992). The importance of EGF in CNS development and preservation is clearly shown by the fact that EGF-mRNA and the membrane-bound EGF receptors (EGFRs) appear very early in mouse embryonic brain and remain in most regions of mouse CNS during adult life (Lazar and Blum 1992). EGFRs have been identified in human (Werner et al 1988) and rat CNS (Gòmez-Pinilla et al. 1988) and are present in neurons (NEUs), astrocytes (ASTs), oligodendrocytes (ODCs) (Mazzoni and Kenigsberg 1992), and microglia (Nolte et al. 1997). Moreover, high EGFR levels have been detected in hippocampal pyramidal cells, Purkinje cells, large NEUs of the dentate nucleus, cells of anterior horn of spinal cord (SC), and cells lining the ventricular system of human developing and adult CNS (Birecree et al. 1991).

The unidirectional penetration of endogenous EGF into the CNS parenchyma through the blood–brain barrier has been reported (Pan and Kastin 1999), and there is indirect evidence of the passage of exogenous EGF (Nakhla and Tam 1985; Scalabrino et al. 1995). The major components of the EGF-EGFR system in mammalian adult CNS and the transport of blood and CNS EGF have been identified. Given that extra˗CNS, EGF˗producing tissues and/or organs (e.g., submaxillary glands, renal tubules, and gastrointestinal Brunner’s glands (Carpenter 1981)) release most of their EGF production in the blood, the effects produced of CNS EGF are actually the sum of both. However, the picture is further complicated by the fact that EGF is also produced by glial cells of the enteric nervous system (Gulbransen and Sharkey 2012).

Only a limited number of reviews have addressed the subject of EGF in the CNS (Fisher and Lakshmanan 1990; Mazzoni and Kenigsberg 1992; Plata-Salamán 1991; Wong and Guillaud 2004; Xian and Zhu 2004; Yamada et al. 1997), the most recent of which was published in 2011 (Gonzalez-Perez and Alvarez-Buylla 2011). Collecting the literature used in this review began at the time of our studies of EGF as a physiological mediator of cobalamin (Cbl) myelinotrophism in human and rat CNS (Scalabrino et al. 1999, 2000), and continued during our studies of CNS EGF levels in multiple sclerosis (MS) (Scalabrino et al. 2010, 2015).

It is widely accepted that the main CNS cell types (ODCs, ASTs, NEUs, and microglia) produce EGF and/or its cognate forms (Mazzoni and Kenigsberg 1992; Plata-Salamán 1991; Scalabrino et al. 1999). Therefore, the effects of EGF on mammalian CNS deserve particular attention for various reasons. First of all, the CNS also has a particular biochemical component called the myelin: this is the predominant non-cell element of white matter, is spiraled extension of the ODC plasma membrane, and is essential for the maintenance and functions of axons and the conduction and propagation of nervous impulses (although the impulse conduction may also be propagated by unmyelinated fibres). Given that the myelin itself has different components (e.g. unique proteins, some glycoproteins, certain classes of lipids, and the tetraspan proteins) (Stadelmann et al. 2019), it must be synthesized, transported, sorted, and assembled in such a way as to ensure the correct molecular architecture (Nave and Trapp 2008; Nave and Werner 2014; Trapp et al. 2004). CNS myelin is an excellent example of the differentiated product assembled by ODCs, although some myelin proteins are also expressed in premyelinating cells (Nave and Werner 2014; Trapp et al. 2004). Many CNS cell types are necessary to build the complex structure of the myelins (Nave and Trapp 2008; Nave and Werner 2014; Trapp et al. 2004; Molina-Gonzalez and Miron 2019) and therefore myelination is one of the best examples of cell–cell cooperativity (Nave and Trapp 2008; Nave and Werner 2014; Trapp et al. 2004). ODCs produce myelin sheaths throughout the CNS and concentrically wrap axons with multi-lamellar sheets of plasma membrane consisting of myelin, but they are also essential for other functions involved in rapid saltatory conduction, such as sodium channel clustering along axonal segments (McTigue and Tripathi 2008), and in metabolic support of axons (Lee et al. 2012). Secondly, neural stem cells (NSCs) are particularly important because they can be expanded and committed to neuronal and neuroglial lineages after suitable stimuli (see “Embryonic, Fetal, and Neonatal CNS Cells” section); this may be especially relevant to the recovery capacity of the CNS after some experimental CNS demyelinating diseases, as well as to MS, which can be considered an epitome of human CNS demyelinating diseases.

The main aim of this review is to describe the studies identifying the effects of EGF on ODCs, their precursors, ASTs and NEUs ( which do not synthesize myelin but are integrated with ODCs in myelinogenesis and myelin maintenance), and microglia. Particular attention is also given to the effects of EGF on NSCs, because this has aroused interest in the potential therapeutic use of EGF in some demyelinating CNS diseases. All of these parts should be understood as being necessary to ensure a better understanding of the EGF role in demyelinating CNS diseases, especially MS, as is the description of the background underlying some aspects of the transfer of remyelination biology from experimental models to the pathogenesis and/or remyelination treatment of MS. Furthermore, given that EGF and its related growth factors of neuregulins (NRGs, proteins sharing an EGF-like signalling domain) (Raabe et al. 1998), heparin-binding (HB)-EGF, amphiregulin, transforming growth factor(TGF)-α (Junier 2000), and β-cellulin (Romano and Bucci 2020), are actually a superfamily of related proteins with similar biological effects, the review will concentrate on “classical” EGF.

Although the effects of EGF obviously occur after its high affinity binding to EGFRs on the cell membrane, the effects of EGFRs themselves on the mammalian CNS will not be discussed as they are beyond the scope of this review. Moreover, the papers indicating the “beneficial” effects of EGF on some experimental non-demyelinating CNS injuries (e.g., hemiparkinsonism, chronic hypoxia, middle cerebral artery occlusion) have not been discussed here, because they have been considered to be out of the focus of interest. For the same reason, the topic of the EGF/EGFR role in some human brain tumors has been deliberately omitted.

## EGF Effects on the Principal Types of CNS Cells

The different effects of EGF on mammalian CNS are here divided on the basis of the different target cells and/or parts of CNS and/or in vitro and/or in vivo methodologies involved, although some of the results have been simultaneously observed in different CNS cell lineages, and/or different CNS parts. The rationale of this is grounded on the well-known notion that CNS myelinogenesis results from a coordinated integration of the main CNS cell phenotypes, although ODCs are the main CNS myelin-forming cells (Traiffort et al. 2020). However, some reported EGF-induced effects on CNS cells are not strictly linked to myelinogenesis, but rather to differentiation and functions of the non-myelin-forming CNS cells, i.e. ASTs, NEUs, and microglia. All together, the studies here reported contribute to give information about one aspect of the capacity and limits of the CNS remyelination. Moreover, the chronological list of the papers dealing with the different effects of EGF on CNS cell neurobiology is also a means of reflecting how CNS-related EGF research has developed over the years.

## Embryonic, Fetal, and Neonatal CNS Cells

It is clearly beyond the scope of this review to discuss the whole field of CNS NSCs, including their fate and their differentiation steps: it would be enough to recall here just some crucial EGF-related points. It is known that cells isolated from some zones of embryonic, neonatal, and adult brain can grow in culture as floating cell clusters (so-called “neurospheres” (NSs)) which are multilineage and capable of self-renewal. It is thought that some of them (including those EGF-generated) are capable of giving rise to the three principal CNS cell phenotypes (i.e., NEUs, ASTs, and ODCs) (Laywell et al. 2000; Reynolds and Weiss 1996), but others appear to be uni- or bi-potential (Vescovi et al. 1993). NSs mainly consist of committed progenitors jumbled with differentiated ASTs and NEUs (Weiss et al. 1996), and have been very useful for demonstrating their capacity to represent the reservoir of NSCs in prenatal and/or adult mammalian CNS (Weiss et al. 1996). EGF-responsive cells also have the capacity to maintain themselves over an extended period of time, another typical characteristic of NSCs (Reynolds and Weiss 1996). NS-forming cells can therefore be considered as NSCs and can act as a potential source of transplantable cells for the treatment of different CNS demyelinating diseases (Doetsch et al. 1999; Ostenfeld et al. 2002). In other words, small NSC populations are present in the developing, newborn, and adult CNS of humans and rodents, some of which can generate cells capable of differentiating into NEUs, ASTs, and ODCs (Laywell et al. 2000; Ostenfeld et al. 2002). At any stage of CNS development, multipotent NSCs can be isolated from various parts of the CNS (Weiss et al. 1996; Doetsch et al. 1999): the subventricular zone (SVZ) adjacent to the striatal wall, the lining of the lateral ventricles, the subgranular zone (SGZ) located at the dentate gyrus within the hippocampus, subcortical white matter, the subcallosal zone located between the corpus callosum and dorsal hippocampus, and SC (Weiss et al. 1996; Seri et al. 2006). Most NSCs are concentrated in the SVZ and the dentate gyrus SGZ (Gage 2000). SVZ and SGZ neural precursors show in vitro clonal expansion leading to NS formation (Recabal et al. 2017).

It is worth noting that EGF (Anton et al. 2004) and EGFR (Gritti et al. 1999; Pastrana et al. 2009) have been detected in vitro and in vivo in SVZ cells in adult rodent brain, even though EGFR + cells of neonatal SVZ are only a small percentage (Cesetti et al. 2009). EGF-responsive cells of the SVZ of the lateral ventricle wall are the source of in vivo EGF-generated NSs (Morshead et al. 1994). EGF has a mitogenic effect on fetal rodent CNS cells, yielding mixed cultures of NEUs and ASTs (Zhu et al. 1999), and it is also a strong mitogen for adult NSCs (Zhu et al. 1999).

The EGF induction of the activity of glutamine synthetase (GS, EC 6.3.1.2, an AST phenotypic marker) has been confirmed in fetal rat telencephalon (also see “Astrocytes” section). Furthermore, the EGF-induced increase in glial fibrillary acidic protein (GFAP, another traditional AST marker)-positive cells has been observed in different parts of rodent embryonic or fetal CNS (see also “Astrocytes” section).

EGF-induced increases in the levels of *Notch-1* intracellular domain in NS cells (Campos et al. 2006) and the cross-talk between *Notch* and EGF in NSCs (Lathia et al. 2008) are of particular interest. Expressed by ODC progenitors (OPCs), ODCs, and ASTs (Wang et al. 1998; Cahoy et al. 2008), *Notch* is a cell surface protein involved in promoting NSC survival, self-renewal, and cell fate choices during CNS development (Lathia et al. 2008), regulates the expression of some transcription factors of the ODC lineage (Nicolay et al. 2007), and directs the differentiation of radial glial cells. Furthermore, *Notch* signalling promotes astroglial differentiation by regulating GFAP gene expression (Ge et al. 2002). Activation of the *Notch* pathway strongly inhibits ODC differentiation and uncouples the relationship between OPC proliferation and differentiation (Wang et al. 1998). Generally speaking, cells with higher levels of *Notch* continue to divide, whereas those with lower levels differentiate. *Notch* has been shown to regulate NSC numbers in vitro and in vivo, and NSCs retain the potential to generate NEUs, ASTs, and ODCs after prolonged exposure to *Notch* ligands (Androutsellis-Theotokis et al. 2006). Furthermore, most *Notch-1* expression in the adult CNS has been demonstrated in SVZ neuroblasts and GFAP-positive SVZ ASTs (Givogri et al. 2006), and the *Notch* pathway cross-talks with *β*1-integrin (Campos et al. 2006) (see also EGF Effects on CNS Extracellular Matrix” section) and EGFR pathway (Aguirre et al. 2010).

It should be remembered that the cellular complexity of NSs (Doetsch et al. 1997) hampers our understanding of their commitment mechanisms, and that they physiologically are simultaneously exposed to different growth factors other than EGF (e.g., platelet derived growth factor, TGF-α, insulin growth factor(IGF) I and II, fibroblast growth factor (FGF), and brain-derived neurotrophic factor), which all influence the NS differentiation programme and some of which have been shown to interact with EGF.

The EGF-related studies dealing with the topic of this section are reported in Table 1.

**Table 1:** Chronological list of the studies assessing the effects of exogenous EGF on different types of cultured cells from different parts of embryonic, fetal, or neonatal mammalian CNSs

Cell source	Effect(s)	Reference
Fetal rat telencephalon	Increase in the levels of CNP- and GS-activity	Guentert-Lauber and Honegger (1983)
Fetal rat telencephalon	Stimulation of the activity of neuronal choline acetyltransferase (EC 2.3.1.6), glutamic acid decarboxylase (EC 4.1.1.15), and glial GS- and CNP- activity	Honegger and Guentert-Lauber (1983)
Fetal rat telencephalon	Stimulation of GS activity and DNA synthesis; increased CNP activity	Guentert-Lauber and Honegger (1985)
Neonatal rat telencephalon	Increase in the number of NEU-like surviving cells; blocked effect by anti-EGF Abs	Morrison et al. (1987)
Fetal rat telencephalon	Increase in GFAP, NEU-specific enolase activity, neurofilaments, and galactocerebroside immunoreactivity	Monnet-Tschudi and Honegger (1989)
Fetal rat telencephalon	Stimulation of cerebellar synthesis of soluble lectins 1 and 2	Tenot et al. (1989)
Embryonic rat mesencephalon	Increase in dopamine and serotonin uptake, and in GFAP-positive cells	Casper et al. (1991)
Embryonic rat mesencephalon	Survival and proliferation of neuronal precursors	Mytilineou et al. (1992)
Embryonic mouse striatum	Induced proliferation of multipotent progenitor cells; generated cells containing several CNS neurotransmitters	Reynolds et al. (1992)
Embryonic mouse striatum	NS formation with the presence of FGF receptor	Vescovi et al. (1993)
Neonatal rat forebrain	Differentiation into NEUs, ASTs, and ODCs; increased GFAP-positive cells	Von Visger et al. (1994)
NEUs from embryonic rat limbic and nonlimbic cortex	Increased LAMP expression in presence of extracellular matrix components (type IV collagen)	Ferri and Levitt (1995)
Embryonic mouse cerebrum	Stimulation of glial precursor proliferation; increased tritiated thymidine incorporation	Kilpatrick and Bartlett (1995)
Embryonic mouse mesencephalon	Cell proliferation towards the NS formation	Santa-Olalla and Covarrubias (1995)
Embryonic rat hippocampus; adult rat forebrain	Expansion of the clones and differentiation into NEUs, ASTs, and ODCs; similar effects with adult subependimal layer cells	Johe et al. (1996)
Embryonic mouse striatum	NS generation; induction of both renewal and expansion of NSCs	Reynolds and Weiss (1996)
NSCs from rat embryo striatum or mesencephalon; human fetal mesencephalon	Expansion of the EGF-responsive cells	Svendsen et al. (1996)
NSCs from mouse embryonic striatum	Formation of NSs which produce NEUs	Schinstine and Iacovitti (1997)
Neonatal mouse hippocampal NSCs	Generation of two types of NSs: one with most NEU-like cells, and the other with most glial-like cells; EGF-responsive cells generate ASTs, NEUs, and ODCs	Shetty and Turner (1998)
Neonatal rat striatum	Formation of NSs and "oligospheres"; EGFR absence in the "oligosphere" cells	Zhang et al. (1998)
Embryonic rat trunk	No mitogenic effect on initially pluripotent (neuroepithelial) cells	Kalyani et al. (1999)
Striatum of embryonic, neonatal, and adult rats	Decreased NS formation (EGF < FGF-2) by increasing age; adult rat SVZ cells are biased more towards neuroglial lineage	Zhu et al. (1999)
Embryonic rat cortex	NS formation; mitotic responsiveness only with the EGFR appearance	Lillien and Raphael (2000)
Embryonic mouse striatal germinal zone	NS generation; identification of an EGF-responsive stem cell population; comparison with the FGF-responsive stem cell population	Martens et al. (2000)
Embryonic mouse cortical ventricular zone cells	NS generation; decreased neurogenic potential and increased gliogenic potential	Qian et al. (2000)
Embryonic mouse striatal SVZ cells	Maintenace of survival and proliferation of neural precursor cells; block of differentiation; no EGF production	Benoit et al. (2001)
Human fetal brain SVZ cells	Generation of a greater number of NSs than that of FGF-generated NSs; the EGF-generated NSs differentiate in ASTs > NEUs > ODCs	Shih et al. (2001)
Newborn rat brain; newborn mouse brain	β1-integrin expression stimulation and MAPK activation in NSs	Campos et al. (2004)
Embryonic and newborn mice	Enhanced *Notch-1* intracellular domain levels in NSs	Campos et al. (2006)
SVZ neurospheres from CNP-EGFR transgenic mice	Increase in EGFR expression and cell proliferation; bias toward ODC fate	Aguirre et al. (2007)
Human fetal brain	Increased growth rate; increased cells with radial glial-like morphology within NSs of neural progenitor cells	Nelson et al. (2008)
NSCs from mouse embryonic telencephalon	Increase in the β1-integrin localization on cell membrane; activated MAPK-signaling pathway; cell proliferation	Suzuki et al. (2010)

## Oligodendrocytes and Their Precursors

EGF has a powerful effect on the fate, amplification and migration of the cells of the SVZ adjacent to the lateral ventricles (also see “Embryonic, Fetal, and Neonatal CNS Cells” section), which express EGFRs (Morshead et al. 1994; Doetsch et al. 2002). ODCs are generated by SVZ cells of the adult mammalian CNS (Menn et al. 2006), which have the definitive characteristics of NSCs (i.e., self-renewal, multipotentiality, and expansion) (Martens et al. 2000). The localized origin of OPCs is not restricted to specific regions of the ventricular zone and the SVZ at particular stages of brain development: OPCs also arise in adult rat SC, which contains glial progenitors that give rise to both ASTs and ODCs (Horner et al. 2000). It has been shown that EGF induces SVZ ASTs to generate OPCs (Gonzalez-Perez and Alvarez-Buylla 2011).

EGF is one of the most powerful epigenetic chemical messengers in determining the fate and maturation of multipotent cells and OPCs. The study showing that ODCs fail to develop in SC explants derived from NRG -/- mice (Vartanian et al. 1999) seems to support this idea, although there is a study with conflict findings (Brinkmann et al. 2008). NEU-derived molecules such as NRGs also act on the proliferation and differentiation of OPCs and/or ODCs, especially during CNS development and therefore are key regulators of CNS myelination (Simons and Trajkovic 2006; McTigue and Tripathi 2008; Kataria et al. 2019). Interestingly, the EGF-like domain of NRGs is necessary and sufficient for the activation of their ErbB receptors (Kataria et al. 2019). EGF has to be considered a powerful promoter because of its positive effects on ODCs and ASTs (which directly and/or indirectly promote myelination (Molina-Gonzalez and Miron 2019)) (Barres and Raff 1999) and on some components of the extra-cellular matrix (ECM) (see “EGF Effects on CNS Extracellular Matrix” section).

ODCs (Raabe et al. 1998; Du and Dreyfus 2002) and OPCs (Peferoen et al. 2014) produce EGF and have EGFRs (Simpson et al. 1982; Peferoen et al. 2014), and so autocrine stimulation can be set up even though both are heterogeneous cell populations (McTigue and Tripathi 2008). There is a negative relationship between proliferation and differentiation in the ODC lineage, because OPCs do not differentiate when dividing, but differentiate into ODCs and myelinate after the withdrawal of mitogens (Wang et al. 1998). Throughout the adult CNS, there is an endogenous pool of OPCs, from which remyelinating ODCs are generated (Gallo and Armstrong 2008).

The EGF induction of 2′,3′-cyclic nucleotide 3′-phosphodiesterase (CNP, EC 3.1.4.37) (also see Table 1) is particularly important, because CNP is widely considered an ODC marker at all stages of myelination (Nave and Trapp 2008). CNP appears early in premyelinating OPCs and remains at high levels as myelin is produced by ODCs (Nave and Trapp 2008). However, specific CNP deletion in mice does not cause any defect in CNS myelination, but causes axonal pathology (Fancy et al. 2011), suggesting that ODCs support axon survival through a myelin-independent mechanism (Lee et al. 2012). It therefore seems that CNP is not essential for myelination, but is required for axonal integrity (Nave and Werner 2014).

As ODCs enter terminal differentiation and contact NEUs, they begin to produce myelin membrane. Therefore, cultured ODCs have the disadvantage that they cannot be used to investigate the axo-glial interactions (Nave and Werner 2014) or some key processes leading to complete myelination: e.g. the match between the number of ODCs and the number of axons to be myelinated (Barres and Raff 1999). Cultured OPCs undergo differentiation, alter their antigenic phenotype, and start to produce myelin components in the absence of NEUs, but OPC development is powerfully controlled by axons (Barres and Raff 1999).

From a point of view of the remyelination capapcity of CNS, nature seems to have favoured the human species in terms of the available reservoir of progenitor cell lines in the normal adult CNS in relation to the feasibility of a possible CNS remyelination, because: (a) NSs and NSCs are abundant, present throughout the CNS, multipotently develop ODCs, ASTs, and NEUs (see “Embryonic, Fetal, and Neonatal CNS Cells” section); (b) OPCs produce the great majority of remyelinating ODCs (Tripathi et al. 2010; Zawadzka et al. 2010) and remain proliferative throughout their life span even in the adult CNS in response to local demyelination and/or injury (Trapp et al. 2004; Moyon et al. 2015); (c) adult OPCs have a transcriptome that is more similar to that of ODCs than that of neonatal OPCs (Moyon et al. 2015); (d) OPCs can give rise to a few remyelinating CNS Schwann cells, that are only partially derived from peripheral nervous system (Franklin and Goldman 2004; Zawadzka et al. 2010); and (e) mature ODCs are still capable of dividing (Trapp et al. 2004; McTigue and Tripathi 2008).

The EGF-related studies dealing with the topic of this section are reported in Table 2.

**Table 2:** Chronological list of the studies assessing the in vitro (A) or in vivo (B) effects of exogenous EGF on ODCs or their progenitors of rodent brain

(A) In vitro		
Cell source	Effect(s)	Reference
Neonatal mouse brain	Inhibition of MBP expression in ODCs; its removal after EGF withdrawal	Sheng et al. (1989)
Adult mouse forebrain	Generation of NSs which give rise to NEUs, ASTs, and ODCs; proliferation of SVZ stem-like cells; EGFR expression	Gritti et al. (1999)
Neonatal rat optic nerve	No stimulation of bromodeoxyuridine incorporation in OPCs	Kondo and Raff (2000a)
Neonatal mouse cerebral cortex	No induction of the formation of new processes or outgrowth of existing processes	Knapp and Adams (2004)
Postnatal brain from CNP/EGFR transgenic mice	Stimulation of the SVZ OPC migration; NEU progenitors and OPCs have both EGF and EGFR; anti-EGF Abs strongly reduce SVZ OPC migration and silence EGFR activation	Aguirre (2005)
Adult mouse brain	Induction of SVZ ASTs (but not the striatal and cortical ASTs) to differentiate towards migratory OPCs and ODCs; coexpression of GFAP and EGFR in SVZ ASTs	Gonzalez-Perez and Quiñones-Hinojosa (2010)

(B) In vivo			
CNS analyzed part	Route of adminstration	Effect(s)	Reference
Postnatal mouse SVZ cells	i.c.v. infusion	Oligodendogenesis promotion from NEU progenitors much greater than that observed in a mutant mouse strain with a hypoactive EGFR	Aguirre and Gallo (2007)
Adult mouse brain SVZ cells	i.c.v. infusion	Proliferation and migration of the SVZ progenitors; differentiation induction of the progenitors along the ODC lineage (OPCs and ODCs)	Gonzalez-Perez et al. (2009)

## Astrocytes

It is worth mentioning that the growth, multiplication, and differentiation of mammalian brain ASTs are under EGF control, although other growth factors are once again involved. ASTs are extremely heterogeneous cell populations (Zhang and Barres 2010; Bayraktar et al. 2014; Khakh and Sofroniew 2015) and play a critical role in both myelination and remyelination of CNS (Molina-Gonzalez and Miron 2019). Furthermore, NSCs sometimes resemble mature ASTs found elsewhere in the brain (Doetsch et al. 1999). Important findings have shown that some SVZ and SGZ ASTs are disguised as NSCs, and are the primary precursors of new NEUs (Doetsch et al. 1999; Ihrie and Alvarez-Buylla 2008).

One key AST function is to support and modulate synaptic functions through reactions that are collectively called the “glutamate-glutamine shuttle” or the “glutamine circle” (Kettenmann and Zorec 2013). In a classical glutamatergic, axo-dendritic synapse, synaptically released glutamate activates neuroglial ionotropic (i) and metabotropic (m) glutamate receptors (GLURs) (Kettenmann and Zorec 2013). Upon glutamate intake, GS (also induced by EGF, see “Embryonic, Fetal, and Neonatal CNS Cells” section) converts it to glutamine, which is then transported back to the NEUs (Kettenmann and Zorec 2013), where it acts as a main source of synthesized glutamate. Given that mGLURs have nearly tenfold higher affinity for glutamate than iGLURs (Morita et al. 2003), the EGF up-regulation of some mGLURs in ASTs also plays a crucial but indirect role in NEU activity, because L-glutamate is the major excitatory chemical neurotransmitter in the mammalian CNS (Kettenmann and Zorec 2013). GLUR activation induces the release of Ca^2+^ from the endoplasmic reticulum Ca^2+^ store. However, it should be borne in mind that: (a) the Ca^2+^ waves differ by astrocytic class and location; and (b) different AST populations differ in their ability to take up extracellular glutamate (Zhang and Barres 2010). It has been postulated that EGF may play a role as a neurotransmitter or neuromodulator in the CNS because of the high-EGF content found in the synaptosomal fractions of mouse cerebral cortex (Lakshmanan et al. 1986) (also see “Neurons and Their Precursors” section). It is therefore not surprising that it has been claimed that NRG-1 is a regulator of both excitatory and inhibitory synaptic transmission in adult brain (Mei and Xiong 2008; Kataria et al. 2019) (also see “Neurons and Their Precursors” and “EGF Effects on CNS Extracellular Matrix” sections). Moreover, the release and signalling of synaptic glutamate also regulate the proliferation of OPCs, and instruct them to differentiate into myelinating ODCs (Gautier et al. 2015).

GFAP is traditionally considered a prototypical astrocytic marker, although: (a) not all ASTs express GFAP and not all cells expressing GFAP in mammalian CNS are ASTs (Morita et al. 2005), and (b) the GFAP gene is only one of the genes whose activation is required for AST differentiation (Oberheim et al. 2012). Reactive astrogliosis also occurs in some CNS demyelinating diseases, such as MS and experimental autoimmune encephalomyelitis (EAE) (Sofroniew 2013). The co-presence of GFAP positivity and EGFR in cultured mouse cerebellar ASTs has been reported (Leutz and Schachner 1982). Although EGF has been included among the molecular regulators and modulators of reactive astrogliosis (Sofroniew 2013), there are conflicting reports on this point: one shows that fetal rat telencephalon GFAP-positive cells increase when EGF is added to the culture medium (Monnet-Tschudi and Honegger 1989), whereas the other shows that GFAP gene expression is lowered in neuroglial cells after EGF exposure (Lazarini et al. 1994). These conflicting data are not surprising because there is not a compulsory parallelism between GFAP positivity and GFAP gene expression in ASTs (Oberheim et al. 2012).

It has been shown in vitro and in vivo that EGF induces the activity of L-ornithine decarboxylase (LOD, EC 4.1.1.17), the rate-limiting enzyme in the polyamine biosynthetic pathway catalyzing the transformation of ornithine into putrescine (Bernstein and Müller 1999). Adult CNS LOD activity is regulated at very low levels, but it is rapidly induced by various physiological CNS stimuli and/or CNS injuries (Bernstein and Müller 1999) (also see Table 4).

*Early growth response(Egr)-1* is localized in the nucleus at a relatively low level, but expressed at high levels in the NEUs of some CNS regions (Beckmann and Wilce 1997). When stimulated by appropriate growth factors such as EGF, NSCs proliferate by activating a multiple signal pathway, that includes the *Ras-Raf-MEK*(MAPK/ERK kinase)*-ERK* (extracellular signal-regulated protein kinase) cascade and is connected with the cell cycle (Gao et al. 2014; Cera et al. 2018). *ERK* activation modulates the expression of several transcription factors including *Egr-1* (Cera et al. 2018). The induction of *Egr-1* by EGF also requires activation of the MAPK (mitogen activated protein kinase) cascade (Mayer et al. 2009). *Egr-1*, *c-fos*, and LOD belong to the family of immediate-early genes, which are also used for CNS activity mapping (Farivar et al. 2004). These genes are activated by different stimuli and link membrane events to the nucleus (Beckmann and Wilce 1997).

All of the EGF family ligands are membrane-anchored precursors and are cleaved by metalloproteases (Eccles 2011). EGFR was found to have kinase activity and consists of an extracellular ligand-binding domain, a transmembrane region, and a cytoplasmatic domain (Eccles 2011). In mouse brain the number of EGFRs has been shown to increase as gestation advanced, whereas their affinities for EGF declined (Adamson and Meek 1984). If EGFR undergoes a low activation, it is subjected to a clathrin-mediate endocytosis, and then it is recycled back to the plsma membrane. If EGFR is highly activated, it is internalized by means of a clathrin-independent endocytosis and then it is degradated in the perinuclear lysosomes (Romano and Bucci 2020). CNS EGFRs play a multifaceted role in: (a) NSC pool maintenance (Aguirre et al. 2010), (b) differentiation, maturation, and morphology of ASTs, (c) ODC differentiation and the consequent increase in the number of myelinating ODCs (Romano and Bucci 2020); (d) axon elongation and dendrite branching (Romano and Bucci 2020), and (e) CNS remyelination after a chemically-induced demyelination (Aguirre et al. 2007). The EGFR signalling pathways after its binding to EGF include the Akt and ERK, which in turn involve MAPK, and JAK/STAT (signal transducers and activators of transcription), phosphatidylinositol 3-kinase pathways, and phospholipase C (Schlessinger 2004; Gao et al. 2014; Romano and Bucci 2020). Akt signalling (the protein kinase B family of serine/threonine protein kinases) is expressed constitutively by ASTs, OPCs, and ODCs, where it is induced by NRG (Flores et al. 2000) and EGF (Codeluppi et al. 2009). This increases CNS myelination by ODCs (Flores et al. 2008) and related myelin sheath thickness, but has no stimulatory power over ODC proliferation (Fancy et al. 2011). Given that Cbl also induces Akt (Okada et al. 2010), Akt is a common step in the signal pathways of EGF, Cbl, and *Notch* (Androutsellis-Theotokis et al. 2006), and IGF-1 (Gaesser and Fyffe-Maricich 2016). This cannot be considered purely casual: transgenic mice with the increased expression of activated Akt in ODCs show marked hypermyelination (Flores et al. 2008). It can be said that Akt signalling participates in many signalling pathways, and is crucial for myelinogenesis, myelin wrapping, and myelin maintenance (Kaplan and Miller 2000).

Some of the effects of EGF on ASTs are also due to the induction of synthesis of IGF-1, which is involved in the positive regulation of OPC differentiation, in mature ODC survival (Franklin and Goldman 2004), and in the differentiation of multipotent adult neuronal progenitor cells into ODCs (Hsieh et al. 2004). It is therefore conceivable that CNS EGF plays its role in the genesis and/or maintenance of CNS myelin by means of different mechanisms, because it: (a) positively regulates proliferation and differentiation of SVZ cells towards a neuroglial lineage (also see “Embryonic, Fetal, and Neonatal CNS Cells” section); (b) directly stimulates astrocytic IGF-1 synthesis; (c) directly stimulates the CNS synthesis of normal cellular prions (PrP^C^s), which are very relevant to the synthesis and maintenance of CNS myelin and are also produced by ODCs (Scalabrino et al. 2012, 2014) (also see Table 4); (d) is the only known physiological mediator of the myelinotrophic effect of Cbl in CNS (Scalabrino et al. 1999, 2000) (also see Table 4); and (e) modulates the OPC Akt activity (also see above) during their terminal differentiation (Mitew et al. 2014). Moreover, there is also an extra-CNS and EGF-linked mechanism by means of which extra-CNS EGF may control and regulate CNS myelin synthesis: EGF is one of the positive regulators of thyroid cell proliferation (Roger et al. 1985), and triiodothyronine plays a major role in controlling the timing of OPC differentiation (Johe et al. 1996), stops the OPC proliferation phase while initiating their terminal differentiation (Roger et al. 1985; Nicolay et al. 2007), and stimulates the expression of several myelin protein genes (Nicolay et al. 2007). Both OPCs and ODCs express thyroid hormone receptor (Barres et al. 1994), and it is no accident that triiodothyronine has been shown to enhance myelin formation by ODCs in SC explants from mouse embryos (Park et al. 2001).

The EGF-related studies dealing with the topic of this section are reported in Table 3.

**Table 3:** Chronological list of the studies assessing the in vitro (A) or in vivo (B) effects of exogenous EGF on CNS ASTs (or neuroglia as a whole)

(A) In vitro		
Cell source	Effect(s)	Reference
Human normal glia	Stimulation of DNA syntesis, ruffling, and macro-pinocytosis	Brunk et al. (1976)
Human brain glia	Increase in cell multiplication and tritiated tymidine incorporation	Westermark (1976)
Neonatal mouse cerebellum	DNA synthesis stimulation; EGFR presence	Leutz and Schachner (1981)
Neonatal mouse cerebellum	Cell GFAP-positivity is accompanied by EGFR presence	Leutz and Schachner (1982)
Rat cerebral hemispheres	Increase in tritiated tymidine incorporation and mitosis of ASTs; EGF-binding in ASTs > ODCs > NEUs	Simpson et al. (1982)
Perinatal rat optic nerve	Stimulation of tritiated thymidine incorporation	Raff et al. (1983)
Neonatal mouse cerebellum	No stimulation of proliferation	Fischer (1984)
Neonatal rat brain	Increase in tritiated thymidine incorporation and mitosis; stimulation of the incorporation of labeled aminoacids in several cytoskeletal proteins	Avola et al. (1988a)
Neonatal rat cerebral cortex	Increase in tritiated thymidine into DNA, and in tritiated uridine into RNA	Avola et al. (1988b)
Rat cerebellum	Increased tritiated thymidine incorporation	Morrison et al. (1988)
Fetal rat hippocampus	Cell proliferation stimulation	Walicke and Baird (1988)
Neonatal rat cerebral hemispheres	Increase in *c-fos* mRNA expression and tritiated thymidine incorporation	Condorelli et al. (1989)
Neonatal rat brain	Stimulation of proliferation and maturation of astroblasts; increased GS activity levels; the mitogenic effect precedes that on cell maturation	Loret et al. (1989)
Neonatal rat brain	Stimulation of tritiated thymidine incorporation into DNA; cell multiplication; specific EGF binding	Wang et al., (1989)
Neonatal rat neocortex	Increased *c-fos* protein immunoreactivity	Hisanaga et al. (1990)
Neonatal rat cerebral cortex	Mitogenic effect; stimulation of 2-deoxyglucose transport and DNA synthesis	Huff and Schreier (1990)
Neonatal rat cerebral cortex	Increased tritiated tymidine incorporation into DNA; increase in 2-deoxyglucose uptake and LOD activity levels	Huff et al. (1990)
Neonatal rat cerebral cortex	Increased FGF-2 release	Araujo and Cotman (1992)
Neonatal rat cerebral cortex	DNA synthesis stimulation; increase in the local IGF-1 synthesis	Han et al., (1992)
Neonatal rat brain	TGF-β1-mRNA up-regulation; mitotic effect	Lindholm et al. (1992)
Neonatal mouse brain	Proliferation and differentiation; down-regulation of GFAP-mRNAs	Lazarini et al. (1994)
Neonatal rat neocortex	Increase in the 1-aminocyclopentane-1,3-dicarboxylic acid-induced hydrolysis of phosphoinositide, and in mGLUR5 levels	Miller et al. (1995)
Aged mouse cerebral hemisphere	Increased GS activity	Grove et al. (1996)
Adult human CNS	Increased tritiated thymidine incorporation; stimulation of cyclins D1, E, A, and B	Liu et al. (1997)
Neocortex of mouse pups	Up-regulation of mGLUR 3 and 5	Minoshima and Nakanishi (1999)
Neonatal rat forebrain	Increased tritiated thymidine incorporation; mitogenic effect (EGF > FGF-2); refractoriness to EGF in aged cultures	Levison et al. (2000)
Adult human cortical glia	Increase in mRNA and interleukin-4 receptor protein	Barna et al. (2001)
Neonatal rat cerebral cortex	Hypertrophic morphology; cell proliferation; Ca^2+^ oscillation due to autocrine activation of EGFR with the consequent Ca^2+^ oscillation; activation of the *Egr-1* promoter; autocrine activation of EGFR	Morita et al. (2005)
Rat optic nerve cells	Increased motility and morphology changes after EGFR activation	Liu et al. (2006)
Neonatal mouse cortex	Phosphorilation of p65, IKKa, and IKKb, activation of Nuclear Factor-kB	Sitcheran et al. (2008)
Adult rat SC	Activation of serine/threonine kinase mTOR pathway; Akt phosphorylartion	Codeluppi et al. (2009)
Neonatal mouse forebrain	*Egr-1* essentiality for the conversion of the mitogenic signal into the proliferative response; transformation of quiescent ASTs in reactive ASTs	Mayer et al. (2009)
Postnatal rodent cortex	Survival promotion	Foo et al. (2011)
Postnatal rat forebrain	Enhancement of cell survival	Scholze et al. (2014)
Postnatal rat SC	Increased nestin expression through Ras-Ref-ERK pathway	Gao et al. (2014)
Mouse cerebral hemispheres	Stimulation of glycogenolysis	Xu et al. (2014)

(B) In vivo			
CNS analyzed part	Route of administration	Effect(s)	Reference
Adult mouse brain SVZ	i.c.v. infusion	Increase in the differentiation and proliferation of NEU precursors towards ASTs; reduced neuroblast number	Doetsch et al. (2002)
Adult mouse SVZ cells	i.c.v. infusion	Stimulation of the in vitro NS generation by SVZ + EGFR-ASTs, a subset of which are activated NSCs	Pastrana et al. (2009)

## Neurons and Their Precursors

A neurotrophic effect (longer cell survival and neurite outgrowth of postnatal cultured rodent striatal, cortical, and cerebral NEUs) has been observed. Furthermore, it is worth noting that the addition of anti-EGF antibodies (Abs) to cultured CNS cells abolishes a number of EGF-induced in vitro effects, thus highlighting the specificity of the EGF effects (also see the anti-EGF Ab effects shown in Tables 1, 2). Repeated intracerebroventricular (i.c.v.) injections of anti-EGF Abs induce myelinolytic lesions in the SC white matter of otherwise normal rats (Scalabrino et al. 2000). These results represent counter-evidence of the importance of EGF in maintaining myelin morphology and structure in the CNS.

Dopamine is an important inuhibitory neurotransmitter in mammalian brain. The EGF-induced increase in dopamine uptake by rodent NEUs seems to be important, because it reinforces the possibility that EGF may also be involved in neurotransmission events (also see “Astrocytes” section). NRGs have been shown to be crucially involved in the modulation of neurotransmission and synaptic plasticity (Kataria et al. 2019; Ledonne and Mercuri 2019).

Adult human brain SVZ cells respond to EGF like those of fetal and/or embryonic CNS cells (Johe et al 1996). This leads to the possibility of expanding these cells into a diseased CNS as a means of restoring functions that have been lost as a result of demyelinating and/or neurodegenerative CNS diseases. Furthermore, in vivo studies based on the administration of exogenous EGF into the lateral ventricles of rodent brain have shown the generation of new NEUs and neuroglia from subependymal stem and progenitor cells. As can also be seen in Table 1, there is a good correspondence between the NSC responses to the in vitro addition of EGF to cultured cells and the in vivo administration of EGF, and between EGF-induced expansion of progenitor cells in the human and rodent CNS (Laywell et al. 2000). The similarities between the EGF-responsive precursors isolated from adult mouse striatum and those isolated from fetal mouse striatum (Reynolds et al. 1992) suggest that these cells represent a persistent reservoir population of NSCs (Weiss et al. 1996).

EGF stimulates *Inhibitor of DNA-binding(Id)1* and inhibits *Id4*, which both belong to the *Id* family of four related helix-loop-helix transcription factors (*Id1*-*Id4*) (Samanta 2004). The fact that EGF up-regulates the level of *Id1* (normally expressed in NSCs) and down-regulates the level of *Id4* (an in vitro inhibitor of oligodendrogenesis) clearly supports the pro-proliferative and pro-differentiative effects of EGF on ODCs, because the decrease in *Id4* corresponds to the time when ODC precursors withdraw from the cell cycle and differentiate (Kondo and Raff 2000b). Moreover, it has been shown that myelin severely inhibits the differentiation of cultured OPCs by means of *Id4* upregulation (Plemel et al. 2013).

The EGF-related studies dealing with the topic of this section are reported in Table 4.

**Table 4:** Chronological list of the studies assessing the effects of exogenous EGF on cultured NEUs and their precursors (A) and on different CNS parts (B)

(A) In vitro		
Cell source	Effect(s)	Reference
Fetal rat telencephalon	Increase in MBP content and LOD- and CNP-activity	Almazan et al. (1985)
NEUs from neonatal rat telencephalon	Enhancement in cell survival and process outgrowth; effect blockage by anti-EGF Abs	Morrison et al. (1987)
NEUs from neonatal rat cerebellum	Enhancement in cell survival and process outgrowth	Morrison et al. (1988)
NEUs from embryonic chick brain	No changes in tritiated thymidine incorporation	Wu et al. (1988)
NEUs from embryonic rat hippocampus	Increase in isoproterenol stimulation of adenylate cyclase and in lactate dehydrogenase (EC 1.1.2.3) activity levels	Pauwels et al. (1989)
NEUs precursors from embryonic chick telencephalon	Powerful neurite-inducing activity; stimulation of ganglioside biosynthesis; effect blockage by anti-EGF Abs	Rosenberg and Noble (1989)
Rat hippocampal slices	Increased magnitude of long-term potentiation after tetanic stimulation	Terlau and Seifert (1989)
NEUs from different regions of embryonic rat CNS	Increased neuronal cell survival	Abe et al. (1990)
Embryonic rat neuroblasts from superior cell ganglia	Increase in tritiated thymidine incorporation and mitosis; DNA synthesis stimulation	DiCicco-Bloom et al. (1990)
NEUs from neonatal rat cortex	Increased cell survival	Kinoshita et al. (1990)
NEUs from embryonic rat mesencephalon	Increased dopamine uptake	Knusel et al. (1990)
NEUs from neonatal rat neocortex	Stimulation of process outgrowth and cell survival	Kornblum et al. (1990)
Adult rat hippocampal slices	Enhanced short-term potentiation and increased magnitude of long-term potentiation	Abe et al. (1991)
NEUs from embryonic rat mesencephalon	Increase in labelled dopamine uptake and GFAP-immunoreactive cells; no effect on the γ-aminobutyric acid (GABA) uptake	Casper et al. (1991)
NEUs from embryonic rat mesencephalon	Increase in the uptake of labelled dopamine and labelled GABA	Ferrari et al. (1991)
NEUs from fetal rat hippocampus	No effect on FGF-2 release	Araujo and Cotman (1992)
Embryonic rat septum	Decreased acetyl cholinesterase (EC 2.3.1.6) activity; abolition of the effect by anti-EGF Abs; increased number of acetylcholinesterase- and GFAP-positive cell number and in the tritiated thymidine incorporation into ASTs but not ODCs	Kenigsberg et al. (1992)
Embryonic rat mesencephalon	Increase in dopamine uptake and neurite outgrowth	Park and Mytilineou (1992)
Undifferentiated EGF-responsive cells from adult mouse striatum	Cell proliferation; differentiation towards NEUs and ASTs; NS formation	Reynolds and Weiss (1992)
Adult mouse brain	Number of neurofilament-positive for EGF-cultures < that for FGF-2-cultures; no stimulation of tritiated thymidine incorporation	Richards et al. (1992)
Fetal rat mesencephalon	Dopamine uptake stimulation	Alexi and Hefti (1993)
Embryonic rat mesencephalon	Increase in dopamine uptake and tritiated thymidine incorporation	Casper et al. (1994)
Neuronal progenitors from germinal layer of murine neonatal cerebellum	Increase in tritiated thymidine incorporation and cell duplication; formation of NS-like multicellular aggregates	Kitchens et al. (1994)
NEUs from embryonic rat SC	Cell proliferation stimulation (FGF-2 > EGF)	Ray and Gage (1994)
Embryonic mouse striatum	*c-fos* induction; generation of NS	Ahmed et al. (1995)
Embryonic rat mesencephalon	Protection from glutamate toxicity in dopaminergic NEUs; increased glutamate uptake	Casper and Blum (1995)
Neuronal precursors from adult mouse striatum	Cell hypertrophia; cell division; NS formation	Gritti et al. (1995)
Mesencephalon and striatum from rat embryos	NS formation from precursor EGF-responsive cells and their maturation in NEUs, ASTs (most), and ODCs (less)	Svendsen et al. (1995)
Different SC regions of adult mouse	NSC expansion only within the lateral ventricles; no proliferation of NEU progenitors located along the borders of third and fourth ventricles	Weiss et al. (1996)
EGF-responsive precursor cells from embryonic rat striatum	Differentiation of most cells to ASTs and a small cell population to NEUs	Rosser et al. (1997)
Embryonic mouse/rat striatum	NS expansion	Svendsen et al. (1997)
Embryonic mouse striatum	Responsiveness to EGF appears later than that to FGF-2; the two growth factors act on the same cell population	Ciccolini and Svendsen (1998)
Embryonic rat hippocampus	Increased number of NEUs and their processes	Peng et al. (1998)
Adult human hippocampus and lateral ventricle wall	NS formation	Johansson et al. (1999)
NSCs from embryonic mouse cortex or striatum	NS formation; EGF-responsiveness appears temporally later than the FGF-2-responsiveness	Tropepe et al. (1999)
Adult rat brain	SVZ cell differentiation into NEUs, ASTs, and ODCs; restriction of EGF-generated neural precursor cells to AST lineage	Whittermore et al. (1999)
Embryonic rat striatum	SVZ cell proliferation, which is mediated by MAPK activation	Learish et al. (2000)
Embryonic mouse striatum	NSC proliferation (NSs), but IGF-1 presence is required	Arsenijevic et al. (2001)
Fetal mouse cortex or striatum	NSC maintenance towards neurogenesis; support of NSC self-renewal	Conti el al. (2005)
Caudate nucleus from adult human post-mortem brain samples	EGF-mRNA presence without age-related changes; EGF transcript levels positively correlate with mRNAs, indicative of immature and differentiated ASTs in human SVZ	Weissleder et al. (2016)
SVZ cells from adult mouse brain	Increased *Egr-1*-expression and -protein; increased cell proliferation; NS induction	Cera et al. (2018)

(B) In vivo		
CNS analyzed part (route of administration)	Effect(s)	Reference
Neonatal mouse brain (subcutaneously)	LOD activity induction and increased tritiated leucine incorporation	Nakhla and Tam (1985)
SC of adult normal or adult Cbl-deficient, hypophysectomized rats (intraperitoneally)	Local LOD activity induction	Scalabrino et al. (1995)
Adult mouse brain (i.c.v.)	Increase in the proliferation of SVZ cells and their migration to the normal brain parenchyma; NS generation; increase in EGFR expression; ASTs > NEUs	Craig et al. (1996)
Adult rat brain (i.c.v.)	Expansion of endogenous SVZ progenitor cells; increase in the number of ASTs, while decreasing that of NEUs; polyp-like hyperplasia of SVZ cells	Kuhn et al. (1997)
Embryonic mouse brain (i.c.v.)	Generation of glial-restricted progenitors from EGF-responsive neural progenitor cells transplanted into the embryonic rat brain	Winkler et al. (1998)
Adult rat brain (i.c.v.)	Increase in the number of bromo-deoxyuridine labelled cells (the polysialylated NEU cell adhesion molecules-immunoreactive) of SVZ; no effect on supraependymal NEU precursors on the surface of third and fourth ventricles	Alonso (1999)
Adult mouse SC (i.c.v.)	No increase in number of bromo-deoxyuridine-labelled nuclei of NSCs around the forth ventricle and in cervical SC	Martens et al. (2002)
Adult mouse brain (intranasal)	Increased SVZ and SGZ neurogenesis; increase in bromodeoxyuridine labeling and in doublecortin-positive cells	Jin et al. (2003)
Adult mouse brain (i.c.v.)	Duplication of striatal SVZ cells into neuroblasts; SVZ cell migration; no maturation of neuroblasts into NEU	Teramoto et al. (2003)
Brain of EGFR-overexpressing adult mouse (i.c.v.)	Neural progenitor cell increase and NSC decrease in the SVZ; *Notch* signaling downregulation; interaction between EGFR- and *Notch*-signaling pathways	Aguirre et al. (2010)
Adult rat brain (i.c.v.)	Enlargement of the SVZ volume and increased cell proliferation; induction of hyperplastic SVZ cells polyps; *Id1* increased and *Id4* decreased levels	Lindberg et al. (2012, 2014)

## Microglia

Few papers dealing with EGF effect on microglia are available and show that microglial cells have EGFR and are stimulated to migrate by EGF, although EGF does not represent a mitotic signal for them (Nolte et al. 1997). Furthermore, EGF triggers a K^+^ conductance in microglia (Ilschner et al. 1996). It should be emphasiszed that specific microglial functions relate to their role in supporting ODC differentiation and meylination (Lloyd and Miron 2019). It is widely known that microglia are also actively involved in clearing myelin debris, which severely hampers CNS remyelination in MS and is one of the great difference between developmental myelination and post-injury CNS remyelination (Neumann et al. 2008). Myelin impairs remyelination in ethidium-bromide-induced demyelination of rat CNS by inhibiting OPC differentiation (Kotter et al. 2006). However, an ODC lineage cell that can phagocytize myelin has been identified (Falcão et al. 2018).

## EGF Effects on CNS Extracellular Matrix

ODC differentiation and myelination are also regulated by ECM-cell interactions, which involve a variety of cell surface molecules (e.g., integrins, cadherins, selectins, and adhesion molecules) (Lau et al. 2013). It is worth noting that EGF increases β1-integrin levels on CNS cell membranes. Integrins play a key role in axo-glial interactions and CNS myelin wrapping through a process depending on Akt (also see “Astrocytes” section) and cross-talk with the *Notch* pathway (Campos et al. 2004, 2006). In particular, ODCs express integrins whose interactions with growth factors regulate the development and number of ODCs in time and space (Baron et al. 2005), and control axonal ensheathment by ODCs (Barros et al. 2009). EGF and integrin signalling play a key role in supporting SVZ progenitor cell migration. Integrins also associate with tetraspanins, cell membrane proteins characterized by four transmembrane domains (Bronstein 2000). It has been shown that HB-EGF binds tetraspanin CD9, which is expressed on CNS myelin and increases in myelinating OPCs (Terada et al. 2002).

The need for environmental cues to ensure correct differentiation and migration of the main CNS cells is emphasized by the finding that EGF interacts with type IV collagen, and this leads to an increase in limbic-system-associated membrane protein (LAMP) levels in cerebral wall progenitors (Ferri and Levitt 1995) (also see Table 1). Given that LAMP is expressed by limbic NEUs but not by non-limbic NEUs in the early phases of cerebral cortical development, EGF regulates the differentiation of non-limbic cell precursors but, when combined with type IV collagen, induces them to express a limbic molecular phenotype.

## EGF and CNS Demyelinating Diseases

### EGF Effects on Experimental Autoimmune Encephalomyelitis Models and Chemically or Virally Induced CNS Demyelination

The different models of EAE and chemically- or virally-induced CNS demyelination are not identical to MS in terms of their histopathological and CSF abnormalities, but they can be considered as mirroring some of the characteristics of MS (Sriram and Steiner 2005; Ransohoff 2012). In addition, the demyelination associated with mouse EAE is largely limited to SC white matter and only later ascends to the brain, whereas MS demyelination first affects both the white and grey matter of the brain and then descends to the SC (Reindl and Waters 2019). Despite these limitations, there is no doubt that EAE models have contributed greatly to our understanding of the phlogistic and cytokine-mediated aspects of MS, as well as the autoimmune-mediated myelin damage (Sriram and Steiner 2005). Finally, EAE models have been used to develop most of the therapeutic approaches to MS, and the approved immunomodulatory drugs have been shown to be effective in treating EAE models (Steinman and Zamvil 2006; Trapp and Nave 2008).

There has been a long-running debate about the role of growth factors in EAE models and chemically- or virally-induced CNS demyelination, also because it is emerging from the literature that the expression of some neurotrophic growth factors other than EGF (e.g., IGFs and FGF) is locally up-regulated in rodent CNS with these CNS demyelinating diseases (McMorris and McKinnon 1996; Hinks and Franklin 1999; Franklin and Hinks 1999; Franklin et al 2001; Armstrong 2007; Gudi et al. 2011). However, the expression of the myelinotrophic growth factors during and/or after induced CNS demyelination is different from that observed during CNS developmental myelination, whereas, ideally, it should be similar (Piaton et al. 2010; Domingues et al. 2016). Although OPCs proceed through the same stages of maturation towards myelinating ODCs during both the developmental myelination and remyelination (Franklin and Hinks 1999; Fancy et al. 2011), the newly formed myelin sheaths are thinner and shorter than normal and their diameters vary much less than during CNS developmental myelination (Hinks and Franklin 1999).

On the basis of our previous results (Scalabrino et al. 1999, 2000, 2010, 2015) and what is known about the effects of EGF on NSCs, differentiation of ODC lineage cells, and the CNS myelin maintenance (see Tables 1, 2, 3, 4), we investigated whether administration of EGF to mice with EAE could improve their SC histopathology as well as their “clinical” picture. We have recently found that the chronic subcutaneous administration of EGF to mice during the development of myelin ODC-specific-glycoprotein(MOG)-induced EAE prevents the onset of the disease and improves the clinical condition and SC histopathology (Nicoletti et al. 2019); furthermore, it has been shown that EGF administration is effective in inducing the CNS remyelination in two other models of chemically-induced CNS demyelination, i.e. lysolecithin-induced and cuprizone-induced CNS demyelination (see Table 5B). Moreover, genetically determined mouse strain with high CNS EGF expression repairs damaged myelin better than a mouse strain with low CNS EGF expression after receiving Theiler's murine encephalomyelitis virus (Bieber et al. 2010). Therefore, the effects of EGF on the different EAE models (which are used as surrogates of MS) and on chemically- or virally-induced CNS demyelination (see Table 5B) are encouraging because, even if administered at pharmacological doses, it appears to be a good remyelination-inducing and demyelination-preventing agent. Given that EGF is classically assigned only a peripheral role in immunology (Klein and Horejsi 1997), it is unlikely that the SC remyelination observed in the EGF-treated EAE mice can be attributed to the possible immunosuppressant and/or antiphlogistic effects of EGF. It is more likely that EGF treatment improved the myelin status and/or structure of SC white matter of the EAE mice and thus cancelled and/or reduced the autoimmune response. In support of this hypothesis, it has been reported that some OPCs in the adult rat CNS are MOG-positive, but lose this immunoreactivity when cultured in the presence of EGF and FGF-2 (Crang et al. 2004). It is also conceivable that EGF treatment induced a stimulus for NSC differentiation towards a glial lineage in the EAE mice. Of note, intranasal HB-EGF administration has been shown to favour SVZ mobilization to lysolecithin-induced demyelination in the adult mouse corpus callosum (Cantarella et al. 2008). NRG has been shown to be effective in diminishing demyelination and enhancing remyelination in SC of mice with myelin basic protein(MBP)-induced EAE (Cannella et al. 1998; Marchionni et al. 1999). Again, intraspinal NRG administration to rats with lysolecithin-lysophosphatidyl-choline-induced SC demyelination promoted differentiation and maturation of local new ODCs and greatly improved endogeneous remyelination (Kataria et al. 2018). These results add credence to a postive role of EGF family for remyelination process in different models of CNS demyelination.

**Table 5:** Chronological list of the studies assessing the in vitro (A) or in vivo (B) effects of exogenous EGF administration in different models of EAE and chemically- or virally-induced CNS demyelination. EGF levels in CNS MS (C)

(A) In vitro			
CNS part	Type of demyelinating drug	Beneficial effect(s)	Reference
Fetal rat telencephalon	anti-MOG Abs	Stimulation of remyelination	Matthieu et al. (1992)
Fetal rat telencephalon	anti-MOG Abs	No changes in EGF mRNA expression	Copelman et al. (2000)

(B) In vivo			
CNS part	Demyelinating agent (route of administration)	Benefical effect(s) (route of administration)	Reference
Mouse corpus callosum	Lysolecithin injection into corpus callosum	Enhanced HB-EGF expression in ipsilateral *vs.* controlateral SVZ cells	Aguirre and Gallo (2007)
Mouse corpus callosum	Lysolecithin injection into corpus callosum	Increase in HB-EGF expression	Aguirre et al. (2007)
Mouse corpus callosum	Lysolecithin injection into corpus callosum	Increase in proliferation, recruitment, and migration of SVZ cells (intranasal HB-EGF administration)	Cantarella et al. (2008)
Mouse corpus callosum	Lysolecithin injection into corpus callosum	Increase in proliferation and migration of SVZ progenitors; most of these cells migrate to the parenchyma around the SVZ and give rise to ODC lineage promoting remyelination (i.c.v. infusion)	Gonzalez-Perez et al. (2009)
Mouse SC	Theiler's murine encephalomyelitis-inducing virus (intracerebral injection)	EGF expression in a highly remyelinating strain much higher than that observed in a non-remyelinating strain	Bieber et al. (2010)
Mouse corpus callosum	Cuprizone-containing diet	Increase in EGF-mRNAs in the corpus callosum and the cortex during both the feeding phase and the remyelination phase	Gudi et al. (2011)
Mouse SC	MOG peptide (subcutaneously)	Improvement in the SC histopathological feature and in clinical score; survival lengthening (intraperitoneal adminstration)	Nicoletti et al. (2019)

(C) Multiple sclerosis			
CNS part		Results	Reference
ASTs in active MS lesions	–	Decreased NRG levels	Viehover et al. (2001)
CSF samples from untreated patients with different clinical courses	–	Decreased levels	Scalabrino et al. (2010)
*Post mortem* CNS samples	–	Decreased SC levels	Scalabrino et al. (2015)
MS patients	–	Increased plasma EGF levels after nutrition with a polysaccaride-based multinutrient diet; coincident with clinical improvement; decreased infection occurrence	Reginald McDaniel et al. (2020)

Taken together, all of the above findings undermine the classical hypothesis that MS is simply an autoimmune CNS disease (Trapp and Nave 2008). However, this is not to minimize the importance of autoimmune and inflammatory reactions in the development of MS or its ominous prognosis, but to indicate that the pathogenesis of MS may equally be based on factors other than autoimmunity (Hemmer et al. 2002; Trapp and Nave 2008).

The EGF- and NRG-related studies dealing with the topic of this section are reported in Table 5A, B.

### EGF in Multiple Sclerosis

As said above, EAE models have fostered the development of new anti-MS drugs, particularly those affecting some aspects of the phlogistic and/or immunological processes in the disease (Hemmer et al. 2002; Franklin and ffrench-Constant 2017). However, the pathogenesis of MS can be investigated differently bearing in mind that: (a) exogenous EGF administration is as effective as Cbl in “curing” myelinolytic lesions in the SC white matter of Cbl-deficient rats without modifying their Cbl-deficient status (Scalabrino et al. 1999, 2000); (b) EGF blocks the in vitro demyelination induced by anti-MOG Abs (see Table 5A); (c) exogeneous EGF administration prevents the EAE onset in mouse SC (Nicoletti et al. 2019); (d) EGF expression is increased in mouse corpus callosum after lysolecithin-induced CNS demyelination (Aguirre et al. 2007); (e) positive Cbl regulation of CSF EGF levels found in Cbl-deficient rats and patients with subacute combined degeneration does not occur in patients whose MS has a different clinical course, because the MS patients have high CSF Cbl levels simultaneously with significantly low CSF EGF levels (Scalabrino et al. 2010); and (f) EGF levels in *post-mortem* SC samples from patients dead with MS (obtained from the Imperial College, London, U.K.) are significantly lower than those of neurological controls dying of non-neurological diseases (Scalabrino et al. 2015). This last finding substantially agrees with that of Viehover et al. (2001) showing that NRG levels are markedly reduced in active MS lesions, although previous studies showed that CSF NRG levels are unchanged in MS patients (Pankonin et al. 2009) and that HB-EGF is overexpressed in ASTs in active MS lesions (Schenk et al. 2013). Other authors (Reginald McDaniel et al. 2020) have found significantly increased blood EGF levels in MS patients after their nutritional status had been optimized and their clinical status improved. It is tempting to speculate that this clinical improvement may have been due to an increase in the CNS production of neurotrophic or myelinotrophic growth factors, because it has been shown that dietary restriction has this effect (Prolla and Mattson 2001).

**Fig. 1:**
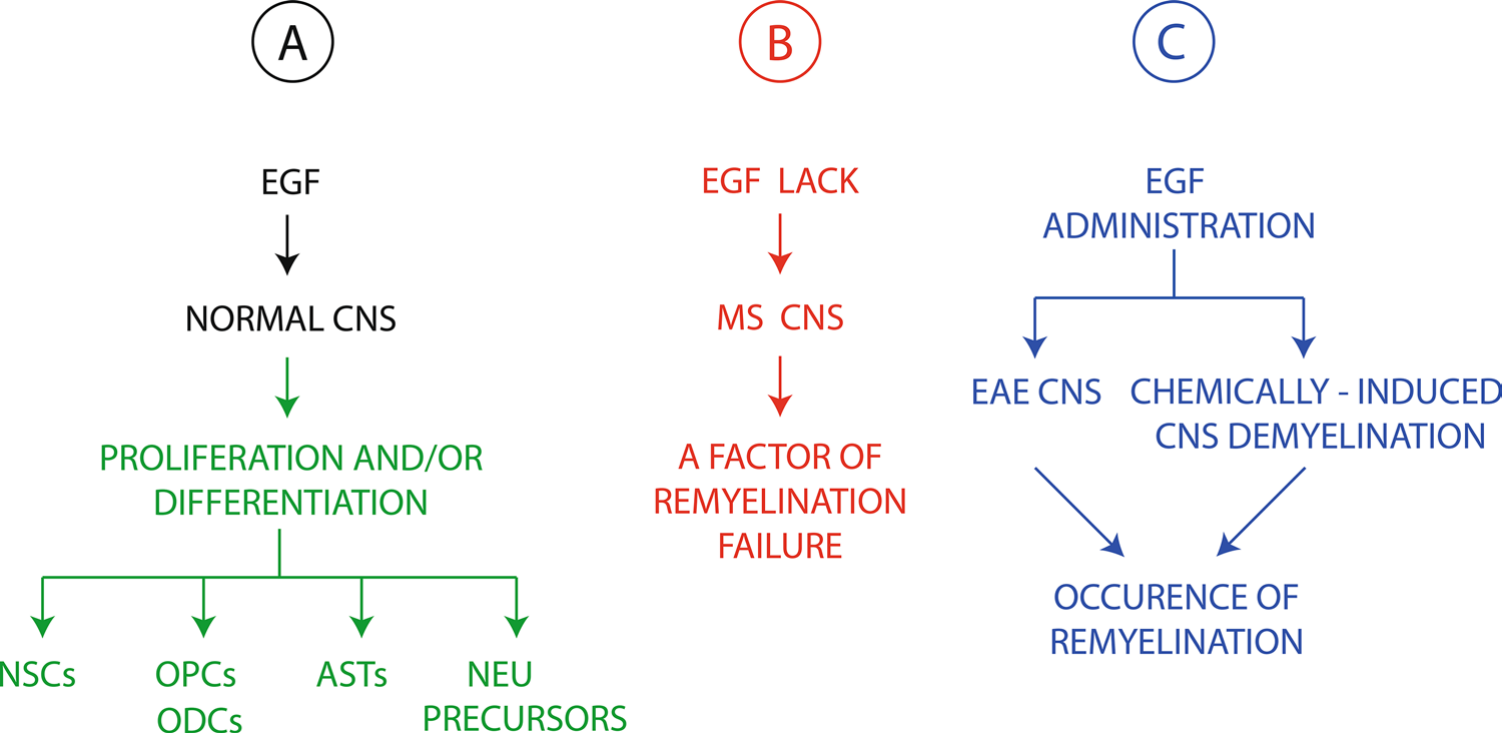
Schematic diagram showing the EGF effects: **a** in normal CNS, **b** in MS CNS, and **c** of its administrations in different experimental models of murine CNS demyelination (see the text for the details and references). Green arrows = increase; red arrows = decrease

**Fig. 2:**
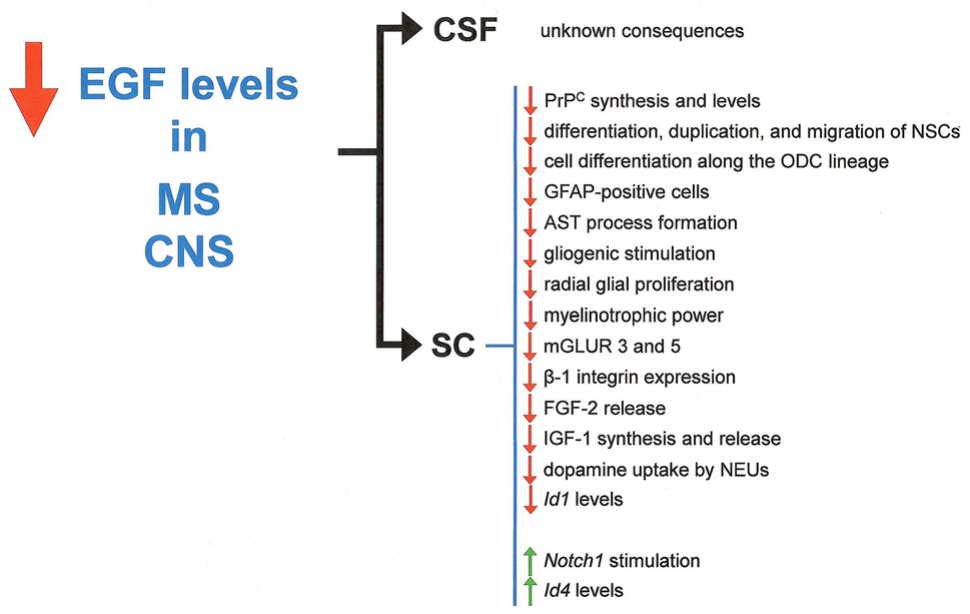
Summary of the possible consequences of EGF deficiency in MS SC. Red arrows, decrease in comparison with normal levels; green arrows, increase in comparison with normal levels (for details and references, see text)

The EGF-related studies dealing with the topic of this section are reported in Table 5C. A schematic diagram depicting: (A) pivotal EGF functions in CNS cell physiology; (B) a key consequence of the EGF lack in MS CNS; and (C) the remyelinating EGF effect in EAE mice and some non-immunological models of rodent CNS demyelination is shown in Fig. 1. Some of the possible negative consequences of CNS EGF deficiency in MS are summarized in Fig. 2, and most of them surely hinder the process of remyelination. Nevertheless, it is exceedingly difficult to assess whether the decreased CSF EGF levels in MS would be specifically involved in MS pathogenesis and/or MS remyelination failure.

### EGF and the Conundrum of Remyelination Failure in Multiple Sclerosis

Some crucial points of the CNS remyelination (directly or indirectly linked to EGF) in adulthood and MS must be emphasized: (a) the SVZ has become a special field of observation for remyelination events in CNS, because SVZ cells proliferate and migrate to various CNS regions, differentiate into mature neuroglia including ODCs, and can be expanded after demyelination damage (Brinkmann et al. 2008) ( also see “Embryonic, Fetal, and Neonatal CNS Cells” section), and the activation of SVZ gliogenesis has been shown in *post-mortem* MS brains and gives rise to OPCs (Nait-Oumesmar et al. 2007); (b) *Notch-1* signalling is expressed and activated in OPCs from *post-mortem* MS brains (Nakahara et al. 2009); (c) *Notch-1* receptor levels are high in ODCs of MS brain (John et al. 2002); (d) the nuclear translocation of *Notch-1*—intracellular domain, required for myelinogenesis, is virtually absent in MS OPCs (Nakahara et al. 2009); (e) OPCs are present in the *post-mortem* MS SC and brain of MS patients (Chang et al. 2000, 2002; Wolswijk 2002), even though it is likely that these OPCs fail to expand or generate new myelin-forming ODCs (Wolswijk 2002; Kuhlmann et al. 2008); and (f) adult OPCs are not identical to perinatal OPCs because they migrate more slowly, have a different cell cycle time, and show different responses to growth factors (McTigue and Tripathi 2008). It is conceivable that the failure of remyelination observed in the majority of chronic MS patients may be multifactorial and due to: (a) a local paucity of ODC lineage precursors, and/or (b) a local excess of OPCs with a fate of apoptotic death, and/or (c) a local deficiency in the neurotrophic growth factors (e.g., EGF) and other molecules (e.g., PrP^c^s and Cbl) essential for the normal OPC differentiation and ODC myelination, and/or (d) differentiation block of OPCs, and/or (e) an increased presence of myelination inhibitors. Even the combination of some of these factors has severe after-effects on the complete MS remyelination process. The picture is further complicated by the fact that MS remyelination is quantitatively quite erratic as it is successful in some patients but not in others (Miller and Mi 2007), and that the remyelination capacity is different in white matter lesions and grey matter lesions.

Furthermore, it is widely known that the local cellular environment of the MS CNS is hostile to remyelination because active ODC remyelination and axon outgrowth is hampered by the local overexpression of some inhibitors (expressed constitutively or after demyelination) of remyelination, such as Nogo, myelin- or ODC-associated glycoprotein (Filbin 2003; Yiu and He 2006), and Leucine-rich repeat and Ig domain-containing Nogo receptor-interacting protein(LINGO)-1. LINGO is selectively expressed by ODCs and NEUs and inhibits OPC and ODC differentiation and myelination (Mi et al. 2008). Notably, LINGO-1 requires EGFR activation (Koprivica et al. 2005). Given that it has been shown that numerous EGFRs are unoccupied and consequently available for EGF ligand in normal CNS (Birecree et al. 1991), it is therefore conceivable that the overwhelming presence of these myelination inhibitors (especially LINGO-1) in MS CNS bind most of the locally available EGFRs, thus preventing the ODC EGFR from binding to the scanty amount of EGF and further reducing new oligodendrogenesis (Aguirre and Gallo 2007).

## Conclusions: Can Discussing EGF Contribute to Broaden the Scenario of the Pathogenesis of Multiple Sclerosis?

Although many studies have recognized demyelination in areas of CNS gray matter (Calabrese et al 2015), MS is generally perceived as a typical white matter disease (Franklin et al. 2001; Trapp and Nave 2008). The traditional neuropathological view of the MS pathogenesis highlights the role of CNS myelin loss, because it leads to the impaired propagation of action potentials across the areas of demyelinated axons and is the major cause of neurological disability. Therefore, the search for mechanisms that primarily cause the demyelination of white matter has always been in the focus of research (Trapp and Nave 2008). It is clear that any treatment is necessarily time-limited and doomed to failure without a precise knowledge of the cause(s) of MS. If MS demyelination is likely caused by a local deficiency in myelinotrophic molecules combined with a local ODC paucity, a local ODC differentiation block, and a local excess of remyelination-impeding agents, it is therefore of primary importance to redress this imbalance by stimulating the search for a means of remyelination: findings showing EGF-, NRG-, and PrP^C^-deficiency in MS SC support this need (Viehover et al. 2001; Scalabrino et al. 2010, 2015). Given that these failures have been observed in *post-mortem* SC samples coming therefore from end-stage MS patients, it is unknown when and why they begin during the course of the disease. Even if myelin repair is well documented during MS course, the major neuropathological lesions found at autopsy are the severely demyelinated plaques, also because the remyelination capacity of MS brain decreases with the disease cronicity (Goldschmidt et al. 2009). Remyelination is thought to be mainly dependent on the local availability of OPCs, which will give rise to nascent remyelinating ODCs. On the assumption that enhancing OPC- and ODC-differentiation in MS lesions could lead to enhance CNS remyelination, regenerative therapies in MS with some myelino- and/or ODC-trophic growth factors have been proposed. For instance, some authors (Patel and Klein 2011; Bankston et al. 2013; Scalabrino et al. 2015; Nicoletti et al. 2019; Villoslada and Steinman 2020) have argued that EGF could theoretically be used (alone or together other myelinotrophic molecules) as a physiological enhancer of CNS remyelination in patients with MS. The possible use of myelinotrophic growth factors (including EGF) to enhance remyelination in MS is supported by papers showing that: (a) several ODC-lineage trophic factors are overexpressed during remyelination after lysolecithin injection in rat CNS (Hinks and Franklin 1999); and (b) a cocktail of many neurotrophic growth factors (other than EGF) promoting the differentiation and proliferation of OPCs and ODCs has been effective in stimulating corpus callosum remyelination in cuprizone-fed mice (Kumar et al. 2007). It is worth noting that intranasal HB-EGF administration immediately after chronic neonatal hypoxia decreases ODC death in CNS mouse and enhances the amount of ODCs from OPCs (Scafidi et al. 2014). It is therefore useful to re-think of the pathogenesis of MS in terms of a re-evaluation of the role of the main myelinotrophic growth factors at the initial phase of the disease, and their correlations between their levels and the degree of neurological disability of the patients. For example, what levels of the main myelinotrophic growth factors might be found in samples of the CSF of the patients with the so-called “benign forms” of MS (Pittock et al 2004)? Therefore, it is still speculative whether EGF levels in MS CNS may serve as an effective tool in the MS diagnosis and/or as an useful biomarker to discriminate the clinical forms of MS, as it has been shown in plasma of MS patients (Tejera-Alhambra et al. 2015).

Many authors have substantially questioned (and even denied) the possibility that MS is primarily an autoimmune disease on the basis of the fact that CNS neurodegeneration precedes autoimmunity (Hemmer et al. 2002; Chaudhuri and Behan 2004; Trapp and Nave 2008; Stys 2010). All together, our findings seem to add further grist to this mill. Furthermore, MS patients treated with appropriate immunosuppressive therapy and/or in an apparent quiescent phase of the disease still show increasing clinical disability and disease progression as the result of remyelination failure (Franklin and Goldman 2004; Franklin and ffrench-Constant 2017). There is no proof-of-principle that MS is an autoimmune disease, and the immune responses to myelin antigens have not been associated with the onset or progression of MS (Hemmer et al. 2002).

Although cellular interactions between ODCs and the other CNS cell types regulate the complex process of remyelination (Molina-Gonzalez and Miron 2019; Traiffort et al 2020), ODCs themselves seem to be ultimately responsible for myelin destruction itself and the failure of MS myelin repair, because: (a) MS MBP is more citrullinated and more deiminated but less phosphorylated than normal MBP (Mastronardi and Moscarello 2005); (b) substantial ODC death has been shown to occur in MS lesions (Barnett and Prineas 2004); (c) the number of OPCs is reduced in chronic MS lesions and they do not mature to ODCs (Kuhlmann et al. 2008); (d) it has recently been shown that ODC death triggers an autoimmune response against CNS myelin in a mouse model, thus suggesting that something similar may happen in MS (Traka et al. 2016); (e) ODC heterogeneity has been recently shown to be altered in *post-mortem* MS samples (Jäkel et al. 2019); (f) recent reports have shown that OPCs and ODCs show transcriptional and epigenomic changes in *post-mortem* MS brains, thus suggesting the possible presence of MS-specific ODCs (Huynh et al. 2014; Falcão et al. 2018); and (g) low levels of ODC expressing transcription factor myelin regulating factor (MYRF, crucial for the OPC maturation into ODCs) have been shown in chronically demyelinated MS lesions (Duncan et al. 2017). Although the production of a wide range of immuno-regulatory factors by ODCs and their role in activating microglia to clear myelin debris out are indisputable (Zeis et al. 2016), it is probably better to look even more carefully at the myelinogenic side of the ODC coin and the NSC potential (Franklin and Gallo 2014), because this may help us to resolve the long-running question as to whether ODCs are culprits or victims in the pathogenesis of MS.

Let me close by quoting a statement of René Descartes taken from “Discours de la methode pour bien conduire sa raison et chercher la verité dans les sciences” (Discourse on the method of rightly conducting one’s reason and of seeking truth in the sciences) (1637; premier livre, premier partie): …la diversité de nos opinions ne vien pas de ce que les uns sont plus raisonables que les autres, mais seulement de ce que nous conduisons nos pensées par diverses voyes et ne considerons pas les mesmes choses” ( … the diversity of our opinions does not arise from some being endowed with a larger share of reason than others, but solely from this, that we conduct our thoughts along different ways, and do not fix our attention on the same objects) (first book, first part). Could the obscurity of the pathogenesis of MS have sparked this debate concerning EGF shedding a small light on a possible new role for myelinotrophic growth factors in MS? But, as Bruce Trapp (2004) cleverly wrote, “the eyes only see what the mind is prepared to comprehend”.

## Data Availability

From the related literature.

## References

[CR1] Abe K, Takayanagi M, Saito H (1990). A comparison of neurotrophic effects of epidermal growth factor and basic fibroblast growth factor in primary cultured neurons from various regions of fetal rat brain. Jpn J Pharmacol.

[CR2] Abe K, Xie F, Saito H (1991). Epidermal growth factor enhances short-term potentiation and facilitates induction of long-term potentiation in rat hippocampal slices. Brain Res.

[CR3] Adamson ED, Meek J (1984). The ontogeny of epidermal growth factor receptors during mouse development. Dev Biol.

[CR4] Aguirre A (2005). Overexpression of the epidermal growth factor receptor confers migratory properties to nonmigratory postnatal neural progenitors. J Neurosci.

[CR5] Aguirre A, Gallo V (2007). Reduced EGFR signaling in progenitor cells of the adult subventricular zone attenuates oligodendrogenesis after demyelination. Neuron Glia Biol.

[CR6] Aguirre A, Dupree JL, Mangin JM (2007). A functional role for EGFR signaling in myelination and remyelination. Nat Neurosci.

[CR7] Aguirre A, Rubio ME, Gallo V (2010). Notch and EGFR pathway interaction regulates neural stem cell number and self-renewal. Nature.

[CR8] Ahmed S, Reynolds B, Weiss S (1995). BDNF enhances the differentiation but not the survival of CNS stem cell-derived neuronal precursors. J Neurosci.

[CR9] Alexi T, Hefti F (1993). Trophic actions of transforming growth factor α on mesencephalic dopaminergic neurons developing in culture. Neuroscience.

[CR10] Almazan G, Honegger P, Matthieu JM (1985). Epidermal growth factor and bovine growth hormone stimulate differentiation and myelination of brain cell aggregates in culture. Brain Res.

[CR11] Alonso G (1999). Neuronal progenitor-like cells expressing polysialylated neural cell adhesion molecule are present on the ventricular surface of the adult rat brain and spinal cord. J Comp Neurol.

[CR12] Androutsellis-Theotokis A, Leker RR, Soldner F (2006). Notch signalling regulates stem cell numbers in vitro and in vivo. Nature.

[CR13] Anton ES, Ghashghaei HT, Weber JL (2004). Receptor tyrosine kinase ErbB4 modulates neuroblast migration and placement in the adult forebrain. Nat Neurosci.

[CR14] Araujo D, Cotman C (1992). Basic FGF in astroglial, microglial, and neuronal cultures: characterization of binding sites and modulation of release by lymphokines and trophic factors. J Neurosci.

[CR15] Armstrong RC (2007). Growth factor regulation of remyelination: behind the growing interest in endogenous cell repair of the CNS. Fut Neurol.

[CR16] Arsenijevic Y, Weiss S, Schneider B (2001). Insulin-like growth factor-I is necessary for neural stem cell proliferation and demonstrates distinct actions of epidermal growth factor and fibroblast growth factor-2. J Neurosci.

[CR17] Avola R, Condorelli DF, Surrentino S (1988). Effect of epidermal growth factor and insulin on DNA, RNA, and cytoskeletal protein labeling in primary rat astroglial cell cultures. J Neurosci Res.

[CR18] Avola R, Condorelli DF, Turpeenoja L (1988). Effect of epidermal growth factor on the labeling of the various RNA species and of nuclear proteins in primary rat astroglial cell cultures. J Neurosci Res.

[CR19] Bankston AN, Mandler MD, Feng Y (2013). Oligodendroglia and neurotrophic factors in neurodegeneration. Neurosci Bull.

[CR20] Barna BP, Mattera R, Jacobs BS (2001). Epidermal growth factor regulates astrocyte expression of the interleukin-4 receptor via a MAPK-independent pathway. Cell Immunol.

[CR21] Barnett MH, Prineas JW (2004). Relapsing and remitting multiple sclerosis: pathology of the newly forming lesion. Ann Neurol.

[CR22] Baron W, Colognato H, ffrench-Constant C (2005). Integrin-growth factor interactions as regulators of oligodendroglial development and function. Glia.

[CR23] Barres BA, Raff MC (1999). Axonal control of oligodendrocyte development. J Cell Biol.

[CR24] Barres BA, Lazar MA, Raff MC (1994). A novel role for thyroid hormone, glucocorticoids and retinoic acid in timing oligodendrocyte development. Development.

[CR25] Barros CS, Nguyen T, Spencer KSR (2009). β1 integrins are required for normal CNS myelination and promote AKT-dependent myelin outgrowth. Development.

[CR26] Bayraktar OA, Fuentealba LC, Alvarez-Buylla A (2014). Astrocyte development and heterogeneity. Cold Spring Harb Perspect Biol.

[CR27] Beckmann AM, Wilce PA (1997). Egr transcription factors in the nervous system. Neurochem Int.

[CR28] Benoit BO, Savarese T, Joly M (2001). Neurotrophin channeling of neural progenitor cell differentiation. J Neurobiol.

[CR29] Bernstein HG, Müller M (1999). The cellular localization of the L-ornithine decarboxylase/polyamine system in normal and diseased central nervous systems. Prog Neurobiol.

[CR30] Bieber AJ, Suwansrinon K, Kerkvliet J (2010). Allelic variation in the Tyk2 and EGF genes as potential genetic determinants of CNS repair. Proc Natl Acad Sci USA.

[CR31] Birecree E, King LE, Nanney LB (1991). Epidermal growth factor and its receptor in the developing human nervous system. Dev Brain Res.

[CR32] Brinkmann BG, Agarwal A, Sereda MW (2008). Neuregulin-1/ErbB signaling serves distinct functions in myelination of the peripheral and central nervous system. Neuron.

[CR33] Bronstein JM (2000). Function of tetraspan proteins in the myelin sheath. Curr Opin Neurobiol.

[CR34] Brunk U, Schellens J, Westermark B (1976). Influence of epidermal growth factor (EGF) on ruffling activity, pinocytosis and proliferation of cultivated human glia cells. Exp Cell Res.

[CR35] Cahoy JD, Emery B, Kaushal A (2008). A transcriptome database for astrocytes, neurons, and oligodendrocytes: a new resource for understanding brain development and function. J Neurosci.

[CR36] Calabrese M, Magliozzi R, Ciccarelli O (2015). Exploring the origins of grey matter damage in multiple sclerosis. Nat Rev Neurosci.

[CR37] Campos LS, Leone DP, Relvas JB (2004). β1 integrins activate a MAPK signalling pathway in neural stem cells that contributes to their maintenance. Development.

[CR38] Campos LS, Decker L, Taylor V (2006). Notch, epidermal growth factor receptor, and β1-integrin pathways are coordinated in neural stem cells. J Biol Chem.

[CR39] Cannella B, Hoban CJ, Gao Y-L (1998). The neuregulin, glial factor 2, diminishes autoimmune demyelination and enhances remyelination in a chronic relapsing model for multiple sclerosis. Proc Natl Acad Sci USA.

[CR40] Cantarella C, Cayre M, Magalon K (2008). Intranasal HB-EGF administration favors adult SVZ cell mobilization to demyelinated lesions in mouse corpus callosum. Dev Neurobiol.

[CR41] Carpenter G, Baserga R (1981). Epidermal growth factor. Tissue growth factors.

[CR42] Casper D, Blum M (1995). Epidermal growth factor and basic fibroblast growth factor protect dopaminergic neurons from glutamate toxicity in culture. J Neurochem.

[CR43] Casper D, Mytilineou C, Blum M (1991). EGF enhances the survival of dopamine neurons in rat embryonic mesencephalon primary cell culture. J Neurosci Res.

[CR44] Casper D, Roboz GJ, Blum M (1994). Epidermal growth factor and basic fibroblast growth factor have independent actions on mesencephalic dopamine neurons in culture. J Neurochem.

[CR45] Cera AA, Cacci E, Toselli C (2018). Egr-1 maintains NSC proliferation and its overexpression counteracts cell cycle exit triggered by the withdrawal of epidermal growth factor. Dev Neurosci.

[CR46] Cesetti T, Obernier K, Bengtson CP (2009). Analysis of stem cell lineage progression in the neonatal subventricular zone identifies EGFR+/NG2- cells as transit-amplifying precursors. Stem Cells.

[CR47] Chang A, Nishiyama A, Peterson J (2000). NG2-positive oligodendrocyte progenitor cells in adult human brain and multiple sclerosis lesions. J Neurosci.

[CR48] Chang A, Tourtellotte WW, Rudick R (2002). Premyelinating oligodendrocytes in chronic lesions of multiple sclerosis. N Engl J Med.

[CR49] Chaudhuri A, Behan PO (2004). Multiple sclerosis is not an autoimmune disease. Arch Neurol.

[CR50] Ciccolini F, Svendsen CN (1998). Fibroblast growth factor 2 (FGF-2) promotes acquisition of epidermal growth factor (EGF) responsiveness in mouse striatal precursor cells: identification of neural precursors responding to both EGF and FGF-2. J Neurosci.

[CR51] Codeluppi S, Svensson CI, Hefferan MP (2009). The Rheb-mTOR pathway is upregulated in reactive astrocytes of the injured spinal cord. J Neurosci.

[CR52] Cohen S (1962). Isolation of a mouse submaxillary gland protein accelerating incisor eruption and eyelid opening in the new-born animal. J Biol Chem.

[CR53] Condorelli DF, Kaczmarek L, Nicoletti F (1989). Induction of protooncogene FOS by extracellular signals in primary glial cell cultures. J Neurosci Res.

[CR54] Conti L, Pollard SM, Gorba T (2005). Niche-independent symmetrical self-renewal of a mammalian tissue stem cell. PLoS Biol.

[CR55] Copelman CA, Cuzner ML, Groome N (2000). Temporal analysis of growth factor mRNA expression in myelinating rat brain aggregate cultures: Increments in CNTF, FGF-2, IGF-I, and PDGF-AA mRNA are induced by antibody-mediated demyelination. Glia.

[CR56] Craig C, Tropepe V, Morshead C (1996). In vivo growth factor expansion of endogenous subependymal neural precursor cell populations in the adult mouse brain. J Neurosci.

[CR57] Crang AJ, Gilson JM, Li WW (2004). The remyelinating potential and in vitro differentiation of MOG-expressing oligodendrocyte precursors isolated from the adult rat CNS. Eur J Neurosci.

[CR58] DiCicco-Bloom E, Townes-Anderson E, Black IB (1990). Neuroblast mitosis in dissociated culture: regulation and relationship to differentiation. J Cell Biol.

[CR59] Doetsch F, Garcìa-Verdugo JM, Alvarez-Buylla A (1997). Cellular composition and three-dimensional organization of the subventricular germinal zone in the adult mammalian brain. J Neurosci.

[CR60] Doetsch F, Caillé I, Lim DA (1999). Subventricular zone astrocytes are neural stem cells in the adult mammalian brain. Cell.

[CR61] Doetsch F, Petreanu L, Caille I (2002). EGF converts transit-amplifying neurogenic precursors in the adult brain into multipotent stem cells. Neuron.

[CR62] Domingues HS, Portugal CC, Socodato R (2016). Oligodendrocyte, astrocyte, and microglia crosstalk in myelin development, damage, and repair. Front Cell Dev Biol.

[CR63] Du Y, Dreyfus CF (2002). Oligodendrocytes as providers of growth factors. J Neurosci Res.

[CR64] Duncan GJ, Plemel JR, Assinck P (2017). Myelin regulatory factor drives remyelination in multiple sclerosis. Acta Neuropathol.

[CR65] Eccles SA (2011). The epidermal growth factor receptor/Erb-B/HER family in normal and malignant breast biology. Int J Dev Biol.

[CR66] Falcão AM, van Bruggen D, Marques S (2018). Diasease-specific oligodendrocyte lineage cells arise in multiple sclerosis. Nat Med.

[CR67] Fallon J, Seroogy K, Loughlin S (1984). Epidermal growth factor immunoreactive material in the central nervous system: location and development. Science.

[CR68] Fancy SPJ, Chan JR, Baranzini SE (2011). Myelin regeneration: A recapitulation of development?. Annu Rev Neurosci.

[CR69] Farivar R, Zangenehpour S, Chaudhuri A (2004). Cellular-resolution activity mapping of the brain using immediate-early gene expression. Front Biosci.

[CR70] Ferrari G, Toffano G, Skaper SD (1991). Epidermal growth factor exerts neuronotrophic effects on dopaminergic and GABAergic CNS neurons: comparison with basic fibroblast growth factor. J Neurosci Res.

[CR71] Ferri RT, Levitt P (1995). Regulation of regional differences in the differentiation of cerebral cortical neurons by EGF family-matrix interactions. Development.

[CR72] Filbin MT (2003). Myelin-associated inhibitors of axonal regeneration in the adult mammalian CNS. Nat Rev Neurosci.

[CR73] Fischer G (1984). Growth requirements of immature astrocytes in serum-free hormonally defined media. J Neurosci Res.

[CR74] Fisher D, Lakshmanan J (1990). Metabolism and effects of epidermal growth factor and related growth factors in mammals. Endocr Rev.

[CR75] Flores AI, Mallon BS, Matsui T (2000). Akt-mediated survival of oligodendrocytes induced by neuregulins. J Neurosci.

[CR76] Flores AI, Narayanan SP, Morse EN (2008). Constitutively active Akt induces enhanced myelination in the CNS. J Neurosci.

[CR77] Foo LC, Allen NJ, Bushong EA (2011). Development of a method for the purification and culture of rodent astrocytes. Neuron.

[CR78] Franklin RJM, ffrench-Constant C (2017). Regenerating CNS myelin—from mechanisms to experimental medicine. Nat Rev Neurosci.

[CR79] Franklin RJM, Gallo V (2014). The translational biology of remyelination: past, present, and future. Glia.

[CR80] Franklin RJM, Goldman JE, Lazzarini R (2004). Remyelination by endogenous glia. Myelin biology and disorders.

[CR81] Franklin RJM, Hinks GL (1999). Understanding CNS remyelination: Clues from developmental and regeneration biology. J Neurosci Res.

[CR82] Franklin RJM, Gillian GL, Voodruff RH (2001). What roles do growth factors play in CNS remyelination?. Progr Brain Res.

[CR84] Gaesser JM, Fyffe-Maricich SL (2016). Intracellular signaling pathway regulation of myelination and remyelination in the CNS. Exp Neurol.

[CR85] Gage FH (2000). Mammalian neural stem cells. Science.

[CR86] Gallo V, Armstrong RC (2008). Myelin repair strategies: a cellular view. Curr Opin Neurol.

[CR87] Gao W-L, Tian F, Zhang S-Q (2014). Epidermal growth factor increases the expression of nestin in rat reactive astrocytes through the Ras-Raf-ERK pathway. Neurosci Lett.

[CR88] Gautier HOB, Evans KA, Volbracht K (2015). Neuronal activity regulates remyelination via glutamate signalling to oligodendrocyte progenitors. Nat Commun.

[CR89] Ge W, Martinowich K, Wu X (2002). Notch signaling promotes astrogliogenesis via direct CSL-mediated glial gene activation. J Neurosci Res.

[CR90] Givogri MI, de Planell M, Galbiati F (2006). Notch signaling in astrocytes and neuroblasts of the adult subventricular zone in health and after cortical injury. Dev Neurosci.

[CR91] Goldschmidt T, Antel J, König FB (2009). Remyelination capacity of the MS brain decreases with disease chronicity. Neurology.

[CR92] Gómez-Pinilla F, Knauer DJ, Nieto-Sampedro M (1988). Epidermal growth factor receptor immunoreactivity in rat brain. Development and cellular localization. Brain Res.

[CR93] Gonzalez-Perez O, Alvarez-Buylla A (2011). Oligodendrogenesis in the subventricular zone and the role of epidermal growth factor. Brain Res Rev.

[CR94] Gonzalez-Perez O, Quiñones-Hinojosa A (2010). Dose-dependent effect of EGF on migration and differentiation of adult subventricular zone astrocytes. Glia.

[CR95] Gonzalez-Perez O, Romero-Rodriguez R, Soriano-Navarro M (2009). Epidermal growth factor induces the progeny of subventricular zone type B cells to migrate and differentiate into oligodendrocytes. Stem Cells.

[CR96] Gritti A, Cova L, Parati EA (1995). Basic fibroblast growth factor supports the proliferation of epidermal growth factor-generated neuronal precursor cells of the adult mouse CNS. Neurosci Lett.

[CR97] Gritti A, Frölichsthal-Schoeller P, Galli R (1999). Epidermal and fibroblast growth factors behave as mitogenic regulators for a single multipotent stem cell-like population from the subventricular region of the adult mouse forebrain. J Neurosci.

[CR98] Grove J, Gomez J, Kentroti S (1996). Plasticity of astrocytes derived from aged mouse cerebral hemispheres: changes with cell passage and immortalization. Brain Res Bull.

[CR99] Gudi V, Škuljec J, Yildiz Ö (2011). Spatial and temporal profiles of growth factor expression during CNS demyelination reveal the dynamics of repair priming. PLoS ONE.

[CR100] Guentert-Lauber B, Honegger P (1983). Epidermal growth factor (EGF) stimulation of cultured brain cells. II. Increased production of extracellular soluble proteins. Dev Brain Res.

[CR101] Guentert-Lauber B, Honegger P (1985). Responsiveness of astrocytes in serum-free aggregate cultures to epidermal growth factor: dependence on the cell cycle and the epidermal growth factor concentration. Dev Neurosci.

[CR102] Gulbransen BD, Sharkey KA (2012). Novel functional roles for enteric glia in the gastrointestinal tract. Nat Rev Gastroenterol Hepatol.

[CR103] Han VKM, Smith A, Myint W (1992). Mitogenic activity of epidermal growth factor on newborn rat astroglia: interaction with insulin-like growth factors. Endocrinology.

[CR104] Hemmer B, Archelos JJ, Hartung HP (2002). New concepts in the immunopathogenesis of multiple sclerosis. Nat Rev Neurosci.

[CR105] Hinks GL, Franklin RJ (1999). Distinctive patterns of PDGF-A, FGF-2, IGF-I, and TGF-β1 gene expression during remyelination of experimentally-induced spinal cord demyelination. Mol Cell Neurosci.

[CR106] Hirata Y, Uchihashi M, Nakajima H (1982). Presence of human epidermal growth factor in human cerebrospinal fluid. J Clin Endocrinol Metab.

[CR107] Hisanaga K, Sagar SM, Hicks KJ (1990). c-fos proto-oncogene expression in astrocytes associated with differentiation or proliferation but not depolarization. Mol Brain Res.

[CR108] Honegger P, Guentert-Lauber B (1983). Epidermal growth factor (EGF) stimulation of cultured brain cells. I. Enhancement of the developmental increase in glial enzymatic activity. Dev Brain Res.

[CR109] Horner PJ, Power AE, Kempermann G (2000). Proliferation and differentiation of progenitor cells throughout the intact adult rat spinal cord. J Neurosci.

[CR110] Hsieh J, Aimone JB, Kaspar BK (2004). IGF-I instructs multipotent adult neural progenitor cells to become oligodendrocytes. J Cell Biol.

[CR111] Huff KR, Schreier W (1990). Fibroblast growth factor inhibits epidermal growth factor-induced responses in rat astrocytes. Glia.

[CR112] Huff KR, Schreier W, Ibric L (1990). Proliferation-related responses in rat astrocytes to epidermal growth factor. Int J Dev Neurosci.

[CR113] Huynh JL, Garg P, Thin TH (2014). Epigenome-wide differences in pathology-free regions of multiple sclerosis-affected brains. Nat Neurosci.

[CR114] Ihrie RA, Alvarez-Buylla A (2008). Cells in the astroglial lineage are neural stem cells. Cell Tissue Res.

[CR115] Ilschner S, Nolte C, Kettenmann H (1996). Complement factor C5a and epidermal growth factor trigger the activation of outward potassium currents in cultured murine microglia. Neuroscience.

[CR116] Jäkel S, Agirre E, Falcão AM (2019). Altered human oligodendrocye heterogeneity in multiple sclerosis. Nature.

[CR117] Jin K, Xie L, Childs J (2003). Cerebral neurogenesis is induced by intranasal administration of growth factors. Ann Neurol.

[CR118] Johansson CB, Svensson M, Wallstedt L (1999). Neural stem cells in the adult human brain. Exp Cell Res.

[CR119] Johe KK, Hazel TG, Muller T (1996). Single factors direct the differentiation of stem cells from the fetal and adult central nervous system. Genes Dev.

[CR120] John GR, Shankar SL, Shafit-Zagardo B (2002). Multiple sclerosis: Re-expression of a developmental pathway that restricts oligodendrocyte maturation. Nat Med.

[CR121] Junier MP (2000). What role(s) for TGFα in the central nervous system?. Prog Neurobiol.

[CR122] Kalyani AJ, Mujtaba T, Rao MS (1999). Expression of EGF receptor and FGF receptor isoforms during neuroepithelial stem cell differentiation. J Neurobiol.

[CR123] Kaplan DR, Miller FD (2000). Neurotrophin signal transduction in the nervous system. Curr Opin Neurobiol.

[CR124] Kataria H, Alizadeh A, Shahriary GM (2018). Neuregulin-1 promotes remyelination and fosters a pro-regenerative inflammatory response in focal demyelinating lesions of the spinal cord. Glia.

[CR125] Kataria H, Alizadeh A, Karimi A (2019). Neuregulin-1/ErbB network: An emerging modulator of nervous system injury and repair. Prog Neurobiol.

[CR126] Kenigsberg RL, Mazzoni IE, Collier B (1992). Epidermal growth factor affects both glia and cholinergic neurons in septal cell cultures. Neuroscience.

[CR127] Kettenmann H, Zorec R, Kettenmann H, Ransom BR (2013). Release of gliotransmitters and transmitter receptors in astrocytes. Neuroglia.

[CR128] Khakh BS, Sofroniew MV (2015). Diversity of astrocyte functions and phenotypes in neural circuits. Nat Neurosci.

[CR129] Kilpatrick T, Bartlett P (1995). Cloned multipotential precursors from the mouse cerebrum require FGF-2, whereas glial restricted precursors are stimulated with either FGF-2 or EGF. J Neurosci.

[CR130] Kinoshita A, Yamada K, Hayakawa T (1990). Modification of anoxic neuronal injury by human recombinant epidermal growth factor and its possible mechanism. J Neurosci Res.

[CR131] Kitchens DL, Snyder EY, Gottlieb DI (1994). FGF and EGF are mitogens for immortalized neural progenitors. J Neurobiol.

[CR132] Klein J, Horejsi V, Klein J, Horejsj V (1997). Cytokines and their receptors. Immunology.

[CR133] Knapp PE, Adams MH (2004). Epidermal growth factor promotes oligodendrocyte process formation and regrowth after injury. Exp Cell Res.

[CR134] Knusel B, Michel P, Schwaber J (1990). Selective and nonselective stimulation of central cholinergic and dopaminergic development in vitro by nerve growth factor, basic fibroblast growth factor, epidermal growth factor, insulin and the insulin-like growth factors I and II. J Neurosci.

[CR135] Kondo T, Raff M (2000). Oligodendrocyte precursor cells reprogrammed to become multipotential CNS stem cells. Science.

[CR136] Kondo T, Raff M (2000). The Id4 HLH protein and the timing of oligodendrocyte differentiation. EMBO J.

[CR137] Koprivica V, Cho KS, Park JB (2005). EGFR activation mediates inhibition of axon regeneration by myelin and chondroitin sulfate proteoglycans. Science.

[CR138] Kornblum HI, Raymon HK, Morrison RS (1990). Epidermal growth factor and basic fibroblast growth factor: effects on an overlapping population of neocortical neurons in vitro. Brain Res.

[CR139] Kotter MR, Li W-W, Zhao C, Franklin RJM (2006). Myelin impairs CNS remyelination by inhibiting oligodendrocyte precursor cell differentiation. J Neurosci.

[CR140] Kuhlmann T, Miron V, Cuo Q (2008). Differentiation block of oligodendroglial progenitor cells as a cause for remyelination failure in chronic multiple sclerosis. Brain.

[CR141] Kuhn HG, Winkler J, Kempermann G (1997). Epidermal growth factor and fibroblast growth factor-2 have different effects on neural progenitors in the adult rat brain. J Neurosci.

[CR142] Kumar S, Biancotti JC, Yamaguchi M (2007). Combination of growth factors enhances remyelination in a cuprizone-induced demyelination mouse model. Neurochem Res.

[CR143] Lakshmanan J, Weichsel ME, Fisher DA (1986). Epidermal growth factor in synaptosomal fractions of mouse cerebral cortex. J Neurochem.

[CR144] Lathia JD, Mattson MP, Cheng A (2008). Notch: from neural development to neurological disorders. J Neurochem.

[CR145] Lau LW, Cua R, Keough MB (2013). Pathophysiology of the brain extracellular matrix: a new target for remyelination. Nat Rev Neurosci.

[CR146] Laywell ED, Rakic P, Kukekov VG (2000). Identification of a multipotent astrocytic stem cell in the immature and adult mouse brain. Proc Natl Acad Sci USA.

[CR147] Lazar L, Blum M (1992). Regional distribution and developmental expression of epidermal growth factor and transforming growth factor-α mRNA in mouse brain by a quantitative nuclease protection assay. J Neurosci.

[CR148] Lazarini F, Castelnau P, Chermann JF (1994). Modulation of prion protein gene expression by growth factors in cultured mouse astrocytes and PC-12 cells. Mol Brain Res.

[CR149] Learish RD, Bruss MD, Haak-Frendscho M (2000). Inhibition of mitogen-activated protein kinase blocks proliferation of neural progenitor cells. Dev Brain Res.

[CR150] Ledonne A, Mercuri NB (2019). On the modulatory roles of neuregulins/ErbB signaling on synaptic plasticity. Int J Mol Sci.

[CR151] Lee Y, Morrison BM, Li Y (2012). Oligodendroglia metabolically support axons and contribute to neurodegeneration. Nature.

[CR152] Leutz A, Schachner M (1981). Epidermal growth factor stimulates DNA-synthesis of astrocytes in primary cerebellar cultures. Cell Tissue Res.

[CR153] Leutz A, Schachner M (1982). Cell type-specificity of epidermal growth factor (EGF) binding in primary cultures of early postnatal mouse cerebellum. Neurosci Lett.

[CR154] Levison SW, Jiang FJ, Stoltzfus OK (2000). IL-6-type cytokines enhance epidermal growth factor-stimulated astrocyte proliferation. Glia.

[CR155] Lillien L, Raphael H (2000). BMP and FGF regulate the development of EGF-responsive neural progenitor cells. Development.

[CR156] Lindberg OR, Brederlau A, Jansson A (2012). Characterization of epidermal growth factor-induced dysplasia in the adult rat subventricular zone. Stem Cells Dev.

[CR157] Lindberg OR, Brederlau A, Kuhn HG (2014). Epidermal growth factor treatment of the adult brain subventricular zone leads to focal microglia/macrophage accumulation and angiogenesis. Stem Cell Rep.

[CR158] Lindholm D, Castrén E, Kiefer R (1992). Transforming growth factor-beta 1 in the rat brain: increase after injury and inhibition of astrocyte proliferation. J Cell Biol.

[CR159] Liu J, Flanagan WM, Drazba JA (1997). The CDK inhibitor, p27Kip1, is required for IL-4 regulation of astrocyte proliferation. J Immunol.

[CR160] Liu B, Chen H, Johns TG (2006). Epidermal growth factor receptor activation: an upstream signal for transition of quiescent astrocytes into reactive astrocytes after neural injury. J Neurosci.

[CR161] Lloyd AF, Miron VE (2019). The pro-remyelination properties of microglia in the central nervous system. Nat Rev Neurol.

[CR162] Loret C, Sensenbrenner M, Labourdette G (1989). Differential phenotypic expression induced in cultured rat astroblasts by acidic fibroblast growth factor, epidermal growth factor, and thrombin. J Biol Chem.

[CR163] Marchionni MA, Cannella B, Hoban C (1999). Neuregulin in neuron/glia interactions in the central nervous system. GGF2 diminishes autoimmune demyelination, promotes oligodendrocyte progenitor expension, and enhances remyelination. Adv Exp Med Biol.

[CR164] Martens DJ, Tropepe V, van der Kooy D (2000). Separate proliferation kinetics of fibroblast growth factor-responsive and epidermal growth factor-responsive neural stem cells within the embryonic forebrain germinal zone. J Neurosci.

[CR165] Martens DJ, Seaberg RM, Van der Kooy D (2002). In vivo infusions of exogenous growth factors into the fourth ventricle of the adult mouse brain increase the proliferation of neural progenitors around the fourth ventricle and the central canal of the spinal cord. Eur J Neurosci.

[CR166] Mastronardi FG, Moscarello MA (2005). Molecules affecting myelin stability: a novel hypothesis regarding the pathogenesis of multiple sclerosis. J Neurosci Res.

[CR167] Matthieu JM, Comte V, Tosic M (1992). Myelin gene expression during demyelination and remyelination in aggregating brain cell cultures. J Neuroimmunol.

[CR168] Mayer SI, Rossler OG, Endo T (2009). Epidermal-growth-factor-induced proliferation of astrocytes requires Egr transcription factors. J Cell Sci.

[CR169] Mazzoni IE, Kenigsberg RL (1992). Effects of epidermal growth factor in the mammalian central nervous system: Its possible implications in brain pathologies and therapeutic applications. Drug Dev Res.

[CR170] McMorris FA, McKinnon RD (1996). Regulation of oligodendrocyte development and CNS myelination by growth factors: prospects for therapy of demyelinating disease. Brain Pathol.

[CR171] McTigue DM, Tripathi RB (2008). The life, death, and replacement of oligodendrocytes in the adult CNS. J Neurochem.

[CR172] Mei L, Xiong WC (2008). Neuregulin 1 in neural development, synaptic plasticity and schizophrenia. Nat Rev Neurosci.

[CR173] Menn B, Garcia-Verdugo JM, Yaschine C (2006). Origin of oligodendrocytes in the subventricular zone of the adult brain. J Neurosci.

[CR174] Mi S, Sandrock A, Miller RH (2008). LINGO-1 and its role in CNS repair. Int J Biochem Cell Biol.

[CR175] Miller RH, Mi S (2007). Dissecting demyelination. Nat Neurosci.

[CR176] Miller S, Romano C, Cotman C (1995). Growth factor upregulation of a phosphoinositide-coupled metabotropic glutamate receptor in cortical astrocytes. J Neurosci.

[CR177] Minoshima T, Nakanishi S (1999). Structural organization of the mouse metabotropic glutamate receptor subtype 3 gene and its regulation by growth factors in cultured cortical astrocytes. J Biochem.

[CR178] Mitew S, Hay CM, Peckham H (2014). Mechanisms regulating the development of oligodendrocytes and central nervous system myelin. Neuroscience.

[CR179] Molina-Gonzalez I, Miron VE (2019). Astrocytes in myelination and remyelination. Neurosci Lett.

[CR180] Monnet-Tschudi F, Honegger P (1989). Influence of epidermal growth factor on the maturation of fetal rat brain cells in aggregate culture. Dev Neurosci.

[CR181] Morita M, Higuchi C, Moto T (2003). Dual regulation of calcium oscillation in astrocytes by growth factors and pro-inflammatory cytokines via the mitogen-activated protein kinase cascade. J Neurosci.

[CR182] Morita M, Kozuka N, Itofusa R (2005). Autocrine activation of EGF receptor promotes oscillation of glutamate-induced calcium increase in astrocytes cultured in rat cerebral cortex. J Neurochem.

[CR183] Morrison R, Kornblum H, Leslie F (1987). Trophic stimulation of cultured neurons from neonatal rat brain by epidermal growth factor. Science.

[CR184] Morrison RS, Keating RF, Moskal JR (1988). Basic fibroblast growth factor and epidermal growth factor exert differential trophic effects on CNS neurons. J Neurosci Res.

[CR185] Morshead CM, Reynolds BA, Craig CG (1994). Neural stem cells in the adult mammalian forebrain: A relatively quiescent subpopulation of subependymal cells. Neuron.

[CR186] Moyon S, Dubessy AL, Aigrot MS (2015). Demyelination causes adult CNS progenitors to revert to an immature state and express immune cues that support their migration. J Neurosci.

[CR187] Mytilineou C, Park TH, Shen J (1992). Epidermal growth factor-induced survival and proliferation of neuronal precursor cells from embryonic rat mesencephalon. Neurosci Lett.

[CR188] Nait-Oumesmar B, Picard-Riera N, Kerninon C (2007). Activation of the subventricular zone in multiple sclerosis: Evidence for early glial progenitors. Proc Natl Acad Sci USA.

[CR189] Nakahara J, Kanekura K, Nawa M (2009). Abnormal expression of TIP30 and arrested nucleocytoplasmic transport within oligodendrocyte precursor cells in multiple sclerosis. J Clin Invest.

[CR190] Nakhla AM, Tam JP (1985). Transforming growth factor is a potent stimulator of testicular ornithine decarboxylase in immature mouse. Biochem Biophys Res Commun.

[CR191] Nave KA, Trapp BD (2008). Axon-glial signaling and the glial support of axon function. Annu Rev Neurosci.

[CR192] Nave KA, Werner HB (2014). Myelination of the nervous system: Mechanisms and functions. Annu Rev Cell Dev Biol.

[CR193] Nelson AD, Suzuki M, Svendsen CN (2008). A high concentration of epidermal growth factor increases the growth and survival of neurogenic radial glial cells within human neurosphere cultures. Stem Cells.

[CR194] Neumann H, Kotter MR, Franklin RJ (2008). Debris clearance by microglia: an essential link between degeneration and regeneration. Brain.

[CR195] Nicolay DJ, Doucette JR, Nazarali AJ (2007). Transcriptional control of oligodendrogenesis. Glia.

[CR196] Nicoletti F, Mazzon E, Fagone P (2019). Prevention of clinical and histological signs of MOG-induced experimental allergic encephalomyelitis by prolonged treatment with recombinant human EGF. J Neuroimmunol.

[CR197] Nolte C, Kirchhoff F, Kettenmann H (1997). Epidermal growth factor is a motility factor for microglial cells in vitro: evidence for EGF receptor expression. Eur J Neurosci.

[CR198] Oberheim NA, Goldman SA, Nedergaard M (2012). Heterogeneity of astrocytic form and function. Methods Mol Biol.

[CR199] Okada K, Tanaka H, Temporin K (2010). Methylcobalamin increases Erk1/2 and Akt activities through the methylation cycle and promotes nerve regeneration in a rat sciatic nerve injury model. Exp Neurol.

[CR200] Ostenfeld T, Joly E, Tai YT (2002). Regional specification of rodent and human neurospheres. Dev Brain Res.

[CR201] Pan W, Kastin AJ (1999). Entry of EGF into brain is rapid and saturable. Peptides.

[CR202] Pankonin MS, Sohi J, Kamholz J (2009). Differential distribution of neuregulin in human brain and spinal fluid. Brain Res.

[CR203] Park TH, Mytilineou C (1992). Protection from 1-methyl-4-phenylpyridinium (MPP+) toxicity and stimulation of regrowth of MPP+-damaged dopaminergic fibers by treatment of mesencephalic cultures with EGF and basic FGF. Brain Res.

[CR204] Park SK, Solomon D, Vartanian T (2001). Growth factor control of CNS myelination. Dev Neurosci.

[CR205] Pastrana E, Cheng LC, Doetsch F (2009). Simultaneous prospective purification of adult subventricular zone neural stem cells and their progeny. Proc Natl Acad Sci USA.

[CR206] Patel JR, Klein RS (2011). Mediators of oligodendrocyte differentiation during remyelination. FEBS Lett.

[CR207] Pauwels PJ, van Assouw HP, Leysen JE (1989). Attenuation of neurotoxicity following anoxia or glutamate receptor activation in EGF- and hippocampal extract-treated neuronal cultures. Cell Signal.

[CR208] Peferoen L, Kipp M, van der Valk P (2014). Oligodendrocyte-microglia cross-talk in the central nervous system. Immunology.

[CR209] Peng H, Wen TC, Tanaka J (1998). Epidermal growth factor protects neuronal cells in vivo and in vitro against transient forebrain ischemia- and free radical-induced injuries. J Cereb Blood Flow Metab.

[CR210] Piaton G, Gould RM, Lubetzki C (2010). Axon-oligodendrocyte interactions during developmental myelination, demyelination and repair. J Neurochem.

[CR211] Pittock SJ, McClelland RL, Mayr WT (2004). Clinical implications of benign multiple sclerosis: a 20-year population-based follow-up study. Ann Neurol.

[CR212] Plata-Salamán CR (1991). Epidermal growth factor and the nervous system. Peptides.

[CR213] Plemel JR, Manesh SB, Sparling JS (2013). Myelin inhibits oligodendroglial maturation and regulates oligodendrocytic transcription factor expression. Glia.

[CR214] Prolla TA, Mattson MP (2001). Molecular mechanisms of brain aging and neurodegenerative disorders: lessons from dietary restriction. Trends Neurosci.

[CR215] Qian X, Shen Q, Goderie SK (2000). Timing of CNS cell generation. Neuron.

[CR216] Raabe TD, Francis A, DeVries GH (1998). Neuregulins in glial cells. Neurochem Res.

[CR217] Raff M, Abney E, Cohen J (1983). Two types of astrocytes in cultures of developing rat white matter: differences in morphology, surface gangliosides, and growth characteristics. J Neurosci.

[CR218] Ransohoff RM (2012). Animal models of multiple sclerosis: the good, the bad and the bottom line. Nat Neurosci.

[CR219] Ray J, Gage F (1994). Spinal cord neuroblasts proliferate in response to basic fibroblast growth factor. J Neurosci.

[CR220] Recabal A, Caprile T, Garcia-Robles MA (2017). Hypothalamic neurogenesis as an adaptive metabolic mechanism. Front Neurosci.

[CR221] Reginald McDaniel H, LaGanke C, Bloom L (2020). The effect of a polysaccharide-based multinutrient dietary supplementation regimen on infections and immune functioning in multiple sclerosis. J Diet Suppl.

[CR222] Reindl M, Waters P (2019). Myelin oligodendrocyte glycoprotein antibodies in neurological disease. Nat Rev Neurol.

[CR223] Reynolds B, Weiss S (1992). Generation of neurons and astrocytes from isolated cells of the adult mammalian central nervous system. Science.

[CR224] Reynolds BA, Weiss S (1996). Clonal and population analyses demonstrate that an EGF-responsive mammalian embryonic CNS precursor is a stem cell. Dev Biol.

[CR225] Reynolds B, Tetzlaff W, Weiss S (1992). A multipotent EGF-responsive striatal embryonic progenitor cell produces neurons and astrocytes. J Neurosci.

[CR226] Richards LJ, Kilpatrick TJ, Bartlett PF (1992). De novo generation of neuronal cells from the adult mouse brain. Proc Natl Acad Sci USA.

[CR227] Roger PP, Heuverswyn B, Lambert C (1985). Antagonistic effects of thyrotropin and epidermal growth factor on thyroglobulin mRNA level in cultured thyroid cells. Eur J Biochem.

[CR228] Romano R, Bucci C (2020). Role of EGFR in the nervous system. Cells.

[CR229] Rosenberg A, Noble EP (1989). EGF-induced neuritogenesis and correlated synthesis of plasma membrane gangliosides in cultured embryonic chick CNS neurons. J Neurosci Res.

[CR230] Rosser AE, Tyers P, ter Borg M (1997). Co-expression of MAP-2 and GFAP in cells developing from rat EGF responsive precursor cells. Dev Brain Res.

[CR231] Samanta J (2004). Interactions between ID and OLIG proteins mediate the inhibitory effects of BMP4 on oligodendroglial differentiation. Development.

[CR232] Santa-Olalla J, Covarrubias L (1995). Epidermal growth factor (EGF), transforming growth factor-α (TGF-α), and basic fibroblast growth factor (bFGF) differentially influence neural precursor cells of mouse embryonic mesencephalon. J Neurosci Res.

[CR233] Scafidi J, Hammond TR, Scafidi S (2014). Intranasal epidermal growth factor treatment rescues neonatal brain injury. Nature.

[CR234] Scalabrino G, Lorenzini EC, Monzio-Compagnoni B (1995). Subacute combined degeneration in the spinal cords of totally gastrectomized rats. Ornithine decarboxylase induction, cobalamin status, and astroglial reaction. Lab Invest.

[CR235] Scalabrino G, Nicolini G, Buccellato FR (1999). Epidermal growth factor as a local mediator of vitamin B12 (cobalamin) in the rat central nervous system. FASEB J.

[CR236] Scalabrino G, Tredici G, Buccellato FR (2000). Further evidence for the involvement of epidermal growth factor in the signaling pathway of vitamin B12 (cobalamin) in the rat central nervous system. J Neuropathol Exp Neurol.

[CR237] Scalabrino G, Galimberti D, Mutti E (2010). Loss of epidermal growth factor regulation by cobalamin in multiple sclerosis. Brain Res.

[CR238] Scalabrino G, Veber D, Mutti E (2012). Cobalamin (vitamin B12) regulation of PrPC, PrPC-mRNA and copper levels in rat central nervous system. Exp Neurol.

[CR239] Scalabrino G, Veber D, Tredici G (2014). Relationships between cobalamin, epidermal growth factor, and normal prions in the myelin maintenance of central nervous system. Int J Biochem Cell Biol.

[CR240] Scalabrino G, Veber D, De Giuseppe R (2015). Low levels of cobalamin, epidermal growth factor, and normal prions in multiple sclerosis spinal cord. Neuroscience.

[CR241] Schenk GJ, Dijkstra S, van het Hof AJ (2013). Roles for HB-EGF and CD9 in multiple sclerosis. Glia.

[CR242] Schinstine M, Iacovitti L (1997). 5-Azacytidine and BDNF enhance the maturation of neurons derived from EGF-generated neural stem cells. Exp Neurol.

[CR243] Schlessinger J (2004). Common and distinct elements in cellular signaling via EGF and FGF receptors. Science.

[CR244] Scholze AR, Foo LC, Mulinyawe S (2014). BMP signaling in astrocytes downregulates EGFR to modulate survival and maturation. PLoS ONE.

[CR245] Seri B, Herrera DG, Gritti A (2006). Composition and organization of the SCZ: a large germinal layer containing neural stem cells in the adult mammalian brain. Cereb Cortex.

[CR246] Sheng HZ, Turnley A, Murphy M (1989). Epidermal growth factor inhibits the expression of myelin basic protein in oligodendrocytes. J Neurosci Res.

[CR247] Shetty AK, Turner DA (1998). In vitro survival and differentiation of neurons derived from epidermal growth factor-responsive postnatal hippocampal stem cells: inducing effects of brain-derived neurotrophic factor. J Neurobiol.

[CR248] Shih CC, Weng Y, Mamelak A (2001). Identification of a candidate human neurohematopoietic stem-cell population. Blood.

[CR249] Simons M, Trajkovic K (2006). Neuron-glia communication in the control of oligodendrocyte function and myelin biogenesis. J Cell Sci.

[CR250] Simpson DL, Morrison R, de Vellis J (1982). Epidermal growth factor binding and mitogenic activity on purified populations of cells from the central nervous system. J Neurosci Res.

[CR251] Sitcheran R, Comb WC, Cogswell PC (2008). Essential role for epidermal growth factor receptor in glutamate receptor signaling to NF-κB. Mol Cell Biol.

[CR252] Sofroniew MV, Kettenmann H, Ransom BR (2013). Astrocyte responses to central nervous system injury and disease. Neuroglia.

[CR253] Sriram S, Steiner I (2005). Experimental allergic encephalomyelitis: a misleading model of multiple sclerosis. Ann Neurol.

[CR254] Stadelmann C, Timmler S, Barrantes-Freer A, Simons M (2019). Myelin in the central nervous system: structure, function, and pathology. Physiol Rev.

[CR255] Steinman L, Zamvil SS (2006). How to successfully apply animal studies in experimental allergic encephalomyelitis to research on multiple sclerosis. Ann Neurol.

[CR256] Stys PK (2010). Multiple Sclerosis: autoimmune disease or autoimmune reaction?. Can J Neurol Sci.

[CR257] Suzuki Y, Yanagisawa M, Yagi H (2010). Involvement of β1-Integrin up-regulation in basic fibroblast growth factor- and epidermal growth factor-induced proliferation of mouse neuroepithelial cells. J Biol Chem.

[CR258] Svendsen CN, Fawcett JW, Bentlage C (1995). Increased survival of rat EGF-generated CNS precursor cells using B27 supplemented medium. Exp Brain Res.

[CR259] Svendsen CN, Clarke DJ, Rosser AE (1996). Survival and differentiation of rat and human epidermal growth factor-responsive precursor cells following grafting into the lesioned adult central nervous system. Exp Neurol.

[CR260] Svendsen CN, Skepper J, Rosser AE (1997). Restricted growth potential of rat neural precursors as compared to mouse. Dev Brain Res.

[CR261] Tejera-Alhambra M, Casrouge A, de Andrés C (2015). Plasma biomarkers discriminate clinical forms of multiple sclerosis. PLoS ONE.

[CR262] Tenot M, Kuchler S, Zanetta JP (1989). Epidermal growth factor enhances the expression of an endogenous lectin in aggregating fetal brain cell cultures. J Neurochem.

[CR263] Terada N, Baracskay K, Kinter M (2002). The tetraspanin protein, CD9, is expressed by progenitor cells committed to oligodendrogenesis and is linked to β1 integrin, CD81, and Tspan-2. Glia.

[CR264] Teramoto T, Qiu J, Plumier JC (2003). EGF amplifies the replacement of parvalbumin-expressing striatal interneurons after ischemia. J Clin Invest.

[CR265] Terlau H, Seifert W (1989). Influence of epidermal growth factor on long-term potentiation in the hippocampal slice. Brain Res.

[CR266] Traiffort E, Kassoussi A, Zahaf A (2020). Astrocyes and microglia as major players of myelin production in normal and pathological conditions. Front Cell Neurosci.

[CR267] Traka M, Podojil JR, McCarthy DP (2016). Oligodendrocyte death results in immune-mediated CNS demyelination. Nat Neuosci.

[CR268] Trapp BD (2004). Pathogenesis of multiple sclerosis: the eyes only see what the mind is prepared to comprehend. Ann Neurol.

[CR269] Trapp BD, Nave KA (2008). Multiple sclerosis: an immune or neurodegenerative disorder?. Annu Rev Neurosci.

[CR270] Trapp BD, Pfeiffer SE, Anitei M, Lazzarini RA (2004). Cell biology of myelin assembly. Myelin biology and disorders.

[CR271] Tripathi RB, Rivers LE, Young KM (2010). NG2 glia generate new oligodendrocytes but few astrocytes in a murine experimental autoimmune encephalomyelitis model of demyelinating disease. J Neurosci.

[CR272] Tropepe V, Sibilia M, Ciruna BG (1999). Distinct neural stem cells proliferate in response to EGF and FGF in the developing mouse telencephalon. Dev Biol.

[CR273] Vartanian T, Fischbach G, Miller R (1999). Failure of spinal cord oligodendrocyte development in mice lacking neuregulin. Proc Natl Acad Sci USA.

[CR274] Vescovi AL, Reynolds BA, Fraser DD (1993). bFGF regulates the proliferative fate of unipotent (neuronal) and bipotent (neuronal/astroglial) EGF-generated CNS progenitor cells. Neuron.

[CR275] Viehover A, Miller RH, Park S-K (2001). Neuregulin: an oligodendrocyte growth factor absent in active multiple sclerosis lesions. Dev Neurosci.

[CR276] Villoslada P, Steinman L (2020). New targets and therapeutics for neuroprotection, remyelination and repair in multiple sclerosis. Expert Opin Investig Drugs.

[CR277] Von Visger JR, Yeon DS, Oh TH (1994). Differentiation and maturation of astrocytes derived from neuroepithelial progenitor cells in culture. Exp Neurol.

[CR278] Walicke PA, Baird A (1988). Neurotrophic effects of basic and acidic fibroblast growth factors are not mediated through glial cells. Dev Brain Res.

[CR279] Wang SL, Shiverick KT, Ogilvie S (1989). Characterization of epidermal growth factor receptors in astrocytic glial and neuronal cells in primary culture. Endocrinology.

[CR280] Wang S, Sdrulla AD, DiSibio G (1998). Notch receptor activation inhibits oligodendrocyte differentiation. Neuron.

[CR281] Weiss S, Dunne C, Hewson J (1996). Multipotent CNS stem cells are present in the adult mammalian spinal cord and ventricular neuroaxis. J Neurosci.

[CR282] Weissleder C, Fung SJ, Wong MW (2016). Decline in proliferation and immature neuron markers in the human subependymal zone during aging: Relationship to EGF- and FGF-related transcripts. Front Aging Neurosci.

[CR283] Werner MH, Nanney LB, Stoscheck CM (1988). Localization of immunoreactive epidermal growth factor receptors in human nervous system. J Histochem Cytochem.

[CR284] Westermark B (1976). Density dependent proliferation of human glia cells stimulated by epidermal growth factor. Biochem Biophys Res Commun.

[CR285] Whittemore SR, Morassutti DJ, Walters WM (1999). Mitogen and substrate differentially affect the lineage restriction of adult rat subventricular zone neural precursor cell populations. Exp Cell Res.

[CR286] Winkler C, Fricker RA, Gates MA (1998). Incorporation and glial differentiation of mouse EGF-responsive neural progenitor cells after transplantation into the embryonic rat brain. Mol Cell Neurosci.

[CR287] Wolswijk G (2002). Oligodendrocyte precursor cells in the demyelinated multiple sclerosis spinal cord. Brain.

[CR288] Wong RWC, Guillaud L (2004). The role of epidermal growth factor and its receptors in mammalian CNS. Cytokine Growth Factor Rev.

[CR289] Wu DK, Maciag T, de Vellis J (1988). Regulation of neuroblast proliferation by hormones and growth factors in chemically defined medium. J Cell Physiol.

[CR290] Xian CJ, Zhu XF (2004). EGF family of growth factors: essential roles and functional redundancy in the nerve system. Front Biosci.

[CR291] Xu J, Song D, Bai Q (2014). Basic mechanism leading to stimulation of glycogenolysis by isoproterenol, EGF, elevated extracellular K+ concentrations, or GABA. Neurochem Res.

[CR292] Yamada M, Ikeuchi T, Hatanaka H (1997). The neurotrophic action and signalling of epidermal growth factor. Prog Neurobiol.

[CR293] Yiu G, He Z (2006). Glial inhibition of CNS axon regeneration. Nat Rev Neurosci.

[CR294] Zawadzka M, Rivers LE, Fancy SPJ (2010). CNS-resident glial progenitor/stem cells produce Schwann cells as well as oligodendrocytes during repair of CNS demyelination. Cell Stem Cell.

[CR295] Zeis T, Enz L, Schaeren-Wiemers N (2016). The immunomodulatory oligodendrocyte. Brain Res.

[CR296] Zhang Y, Barres BA (2010). Astrocytes heterogeneity: an underappreciated topic in neurobiology. Curr Opin Neurobiol.

[CR297] Zhang SC, Lundberg C, Lipsitz D (1998). Generation of oligodendroglial progenitors from neural stem cells. J Neurocytol.

[CR298] Zhu G, Mehler MF, Mabie PC (1999). Developmental changes in progenitor cell responsiveness to cytokines. J Neurosci Res.

